# Molecular Determinants of Intravitreal Anti-VEGF Durability: Drug Architecture, Intraocular Pharmacokinetics, Target Biology and Treatment Resistance

**DOI:** 10.3390/ijms27177786

**Published:** 2026-08-31

**Authors:** Georgios D. Panos, Theo Empeslidis, Efstratia Amaxilati, Georgios N. Tsiropoulos, Nikolaos Topouzis, Panagiotis A.G. Konstas, Eleftherios Chatzimichail, Zisis Gatzioufas, Winfried Amoaku

**Affiliations:** 1 Division of Ophthalmology and Visual Sciences, School of Medicine, University of Nottingham, Nottingham NG7 2UH, UK; 2 First Department of Ophthalmology, AHEPA University Hospital, School of Medicine, Aristotle University of Thessaloniki, 54636 Thessaloniki, Greece; 3 Vantage Biosciences Ltd., London SW1Y 5ES, UK; 4 Department of Ophthalmology, School of Medicine, University of Ioannina, 45110 Ioannina, Greece; 5 Department of Ophthalmology, University Hospital of Basel, 4056 Basel, Switzerland

**Keywords:** anti-VEGF, ocular pharmacokinetics, durability, retinal vascular disease, target suppression, sustained delivery, gene therapy

## Abstract

Intravitreal inhibition of vascular endothelial growth factor (VEGF) has transformed the management of neovascular age-related macular degeneration, diabetic macular oedema and macular oedema secondary to retinal vein occlusion, but frequent monitoring and retreatment remain major burdens. Ocular half-life is often used as shorthand for durability, although the clinical interval is produced by a wider molecular and biological system. This narrative review examines how dose, molecular format, hydrodynamic size, binding affinity, valency, ligand spectrum, target turnover, tissue distribution and delivery architecture determine the time for which an eye remains controlled. Human ocular pharmacokinetic and pharmacodynamic evidence is interpreted according to compartment, assay and model provenance, with particular attention to the distinction between drug elimination, free-ligand suppression, anatomical control and protocol-assigned treatment interval. Trial evidence for ranibizumab, aflibercept, conbercept, brolucizumab and faricimab shows that extended dosing can arise from greater starting exposure, altered binding architecture or pathway expansion without a proportionate change in intrinsic ocular half-life. Patient phenotype and retreatment rules further modify the observed interval. Refillable reservoirs, biodegradable depots and ocular gene therapy change the governing kinetics from bolus elimination to controlled release or sustained local production, thereby increasing the importance of reversibility and cumulative safety. We propose that durability be defined as a time-to-threshold phenotype integrating active target-site exposure, biological demand, anatomical recurrence, all treatment-related procedures and safety. Standardised estimands, longitudinal human ocular sampling, spatial exposure methods and externally validated mechanism-informed models are needed to make molecular durability comparable and clinically actionable.

## 1. Introduction

Intravitreal inhibition of vascular endothelial growth factor (VEGF) has changed the natural history of neovascular age-related macular degeneration (nAMD), diabetic macular oedema (DMO) and macular oedema secondary to retinal vein occlusion (RVO). Pivotal trials established that repeated VEGF-A neutralisation can preserve or improve vision across these diseases [1,2,3,4,5]. In addition, inhibition of complementary pathways, including angiopoietin-2, can modify vascular stability. Despite this success, treatment remains a chronic-care challenge: benefit depends on maintaining sufficient intraocular drug activity, while repeat injections, monitoring visits, travel, anxiety and demands on service capacity accumulate over years.

Durability is commonly discussed as if it were an intrinsic molecular constant. In practice, the word is used for several non-equivalent quantities: ocular elimination half-life, duration of free-ligand suppression, time to recurrent retinal fluid, maximum interval permitted by a protocol, interval achieved by an individual eye and freedom from any supplemental procedure. These measures are linked, but none can be inferred reliably from another without assumptions. A product can provide a longer clinical interval because its activity begins at a higher molar concentration, binds its target differently, engages an additional pathway or is delivered continuously—even if its intrinsic elimination rate is unchanged.

The molecular question is, therefore, not simply how long a drug remains measurable in the vitreous. The clinically relevant question is how long the active drug remains available at the retina–retinal pigment epithelium–choroid interface at a concentration sufficient to control the disease process. That interval is affected by molecular mass, hydrodynamic radius, charge, binding stoichiometry, target abundance, complex formation, tissue barriers and clearance. It is then modified by lesion phenotype, inflammatory and ischaemic signals, anatomical tolerance rules and the assessment schedule. The broad success of anti-VEGF therapy and the subsequent diversification of molecular formats provide a natural experiment in how these determinants interact [6].

**Figure 1:**
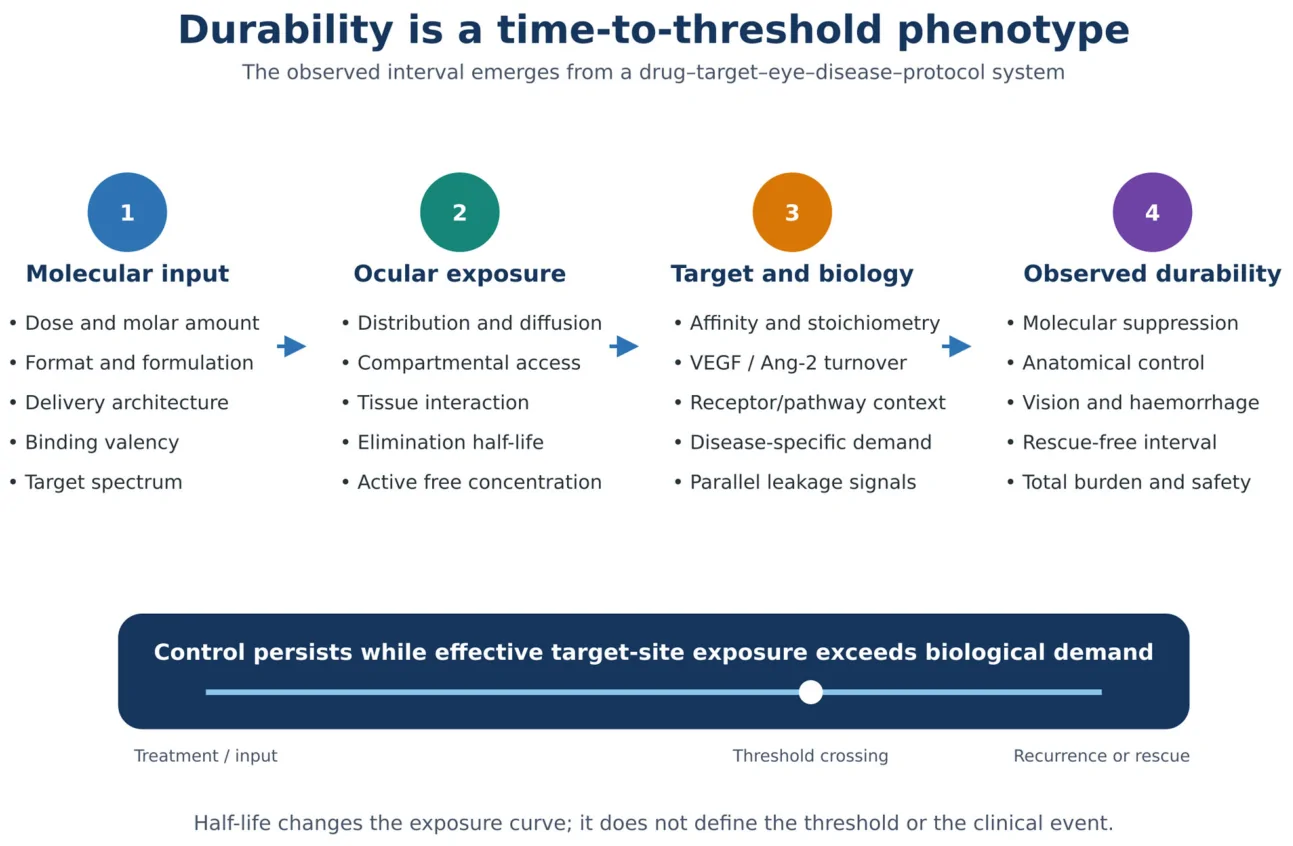
Durability as a time-to-threshold phenotype. The observed clinical interval results from the interaction of molecular input and distribution, ligand binding and target suppression, eye- and disease-specific biological demand, and protocol-defined recurrence or rescue. Half-life is one determinant within this system rather than a synonym for durability. Ang-2, angiopoietin-2; VEGF, vascular endothelial growth factor.

This review develops a molecular framework for interpreting durability across bolus anti-VEGF biologics and emerging sustained-exposure platforms. It first distinguishes the relevant durability constructs, then examines VEGF and angiopoietin biology, molecular architecture, human ocular pharmacokinetics and target suppression, clinical trial translation, patient-level modifiers, emerging delivery strategies and research priorities. Durability is, therefore, treated as a time-to-threshold phenotype generated by a drug–target–eye–disease–protocol system (Figure 1).

## 2. Scope, Terminology and Narrative Review Approach

### 2.1. Scope and Literature Approach

This article is a narrative review of molecular and translational determinants of anti-VEGF durability in retinal vascular disease. PubMed/MEDLINE and Scopus searches were updated through 18 August 2026. The complete database-specific core searches were as follows:

PubMed/MEDLINE: ((“Vascular Endothelial Growth Factors”[Mesh] OR “Vascular Endothelial Growth Factor A”[Mesh] OR VEGF[Title/Abstract] OR “anti-VEGF”[Title/Abstract] OR ranibizumab[Title/Abstract] OR bevacizumab[Title/Abstract] OR aflibercept[Title/Abstract] OR brolucizumab[Title/Abstract] OR faricimab[Title/Abstract] OR conbercept[Title/Abstract]) AND (intravitreal[Title/Abstract] OR ocular[Title/Abstract] OR retina*[Title/Abstract] OR “macular degeneration”[Mesh] OR “neovascular age-related macular degeneration”[Title/Abstract] OR “diabetic macular edema”[Title/Abstract] OR “diabetic macular oedema”[Title/Abstract] OR “retinal vein occlusion”[Title/Abstract]) AND (durab*[Title/Abstract] OR pharmacokinetic*[Title/Abstract] OR pharmacodynamic*[Title/Abstract] OR “half-life”[Title/Abstract] OR “target suppression”[Title/Abstract] OR “molecular size”[Title/Abstract] OR “binding affinity”[Title/Abstract] OR “treatment interval”[Title/Abstract] OR “treat and extend”[Title/Abstract] OR implant*[Title/Abstract] OR depot[Title/Abstract] OR “sustained delivery”[Title/Abstract] OR “gene therapy”[Title/Abstract] OR angiopoietin-2[Title/Abstract] OR Tie2[Title/Abstract] OR safety[Title/Abstract] OR adverse event*[Title/Abstract] OR inflammation[Title/Abstract] OR vasculitis[Title/Abstract] OR cost*[Title/Abstract] OR pharmacoeconomic*[Title/Abstract] OR cost-effectiveness[Title/Abstract])).

Scopus: TITLE-ABS-KEY((VEGF OR “anti-VEGF” OR ranibizumab OR bevacizumab OR aflibercept OR brolucizumab OR faricimab OR conbercept) AND (intravitreal OR ocular OR retina* OR “macular degeneration” OR “neovascular age-related macular degeneration” OR “diabetic macular edema” OR “diabetic macular oedema” OR “retinal vein occlusion”) AND (durab* OR pharmacokinetic* OR pharmacodynamic* OR “half-life” OR “target suppression” OR “molecular size” OR “binding affinity” OR “treatment interval” OR “treat and extend” OR implant* OR depot OR “sustained delivery” OR “gene therapy” OR angiopoietin-2 OR Tie2 OR safety OR “adverse event*” OR inflammation OR vasculitis OR cost* OR pharmacoeconomic* OR cost-effectiveness)).

Reference lists of key articles were reviewed, and trial registries, regulatory guidance and sponsor reports were consulted selectively for emerging programmes not yet fully represented in the peer-reviewed literature. Searches were not restricted by a formal start date. The search was iterative and consistent with the narrative objective; no PRISMA-style study count is implied.

Because the objective was interpretive synthesis rather than exhaustive effect estimation, detailed PRISMA-style eligibility criteria and formal risk-of-bias scoring were not applied. Priority was given to direct human ocular concentration or pharmacodynamic measurements, prospectively defined comparative trials, mechanistically informative preclinical work and transparent pharmacokinetic–pharmacodynamic models. English-language sources with clear methodological provenance were emphasised; older foundational studies and current development reports were included, when necessary, to explain molecular design or development status. The review was planned and critically revised with reference to the SANRA framework for narrative review quality, including justification of importance, statement of aims, description of the literature search, referencing, scientific reasoning and presentation of relevant endpoint data [7].

### 2.2. Operational Definitions

Table 1 defines the durability constructs used throughout this review. Molecular persistence refers to drug concentration in a specified ocular compartment; molecular pharmacodynamic durability refers to target suppression; anatomical durability refers to maintenance of prespecified imaging control; and clinical durability refers to a prospectively defined interval without rescue. Delivery durability describes the exposure profile produced by a device, depot or transgene, whereas net durability integrates efficacy with every associated procedure, monitoring demand and safety event. These terms should be reported separately before they are combined.

## 3. Biological Basis of Anti-VEGF Durability

### 3.1. VEGF Ligands, Receptors and Disease Demand

The VEGF family comprises ligands with overlapping but non-identical receptor interactions. VEGF-A is the dominant therapeutic target in retinal vascular disease and signals principally through VEGFR-2 to promote endothelial permeability, proliferation, survival and pathological angiogenesis; VEGFR-1 modulates ligand availability and cellular responses [8]. Placental growth factor (PlGF) and VEGF-B bind VEGFR-1, while VEGF-C and VEGF-D can engage VEGFR-2 after proteolytic processing. The biological demand placed on a therapeutic molecule, therefore, reflects not only VEGF-A concentration but also ligand isoforms, extracellular-matrix binding, receptor density and the contribution of parallel family members.

VEGF-A binding to VEGFR-2 initiates several intracellular programmes rather than a single angiogenic signal. PLCγ–PKC–Raf–MEK–ERK signalling supports endothelial proliferation, whereas PI3K–AKT/eNOS promotes survival, migration and nitric-oxide responses. Src-family, focal-adhesion and cytoskeletal pathways destabilise VE-cadherin-containing junctions and increase permeability [6,8]. Neutralisation of extracellular VEGF, therefore, suppresses leakage and neovascular growth but does not imply uniform or synchronous intracellular suppression across endothelial cells, retinal pigment epithelium, Müller cells and mural cells.

The consequences of prolonged suppression for the vascular wall require careful qualification. Pericyte recruitment and maintenance depend principally on endothelial PDGF-B/PDGFRβ signalling, with VEGF-related effects occurring through reciprocal endothelial–pericyte crosstalk rather than a simple direct survival requirement [9]. The endothelial glycocalyx regulates permeability, mechanotransduction and the presentation of heparan-sulphate-bound growth factors; diabetes and inflammation can disrupt it, but chronic intravitreal anti-VEGF therapy has not been shown to cause glycocalyx loss in human retinal circulation [10]. VEGF-responsive microRNAs, including the miR-15a/16 family, can modulate PI3K–AKT, junctional proteins and endothelial barrier behaviour in experimental systems [11]; however, no microRNA signature currently predicts a safe treatment interval.

Complete genetic or continuous systemic VEGF deprivation in adult animals can injure neural retinal cells or the choriocapillaris, supporting a physiological trophic role for endogenous VEGF [12,13]. Such models are not equivalent to partial, intermittent intravitreal neutralisation at clinical doses. Long-term effects on pericyte function, glycocalyx integrity and microRNA networks are, therefore, biologically plausible research questions, not established adverse consequences of current ocular treatment.

Ligand production and elimination are dynamic. Hypoxia-inducible signalling, inflammation, oxidative stress, advanced glycation, mechanical stress and macrophage activity can increase VEGF synthesis in retinal pigment epithelium, Müller cells, vascular endothelium and infiltrating cells. A molecule eliminated at the same rate in two eyes can consequently cross the effective threshold at different times because the rate of VEGF replenishment differs. High ligand production also increases target-mediated binding and complex formation, potentially changing the fraction of free active drug, even when total drug concentrations appear similar.

Disease phenotype determines where this demand is expressed. In nAMD, the relevant compartment may be subretinal, sub-retinal-pigment-epithelial or intraretinal and may include mature vessels, fibrosis and macrophage-rich lesions. In DMO, diffuse blood–retinal-barrier failure, inflammation and systemic metabolic factors can coexist. In RVO, abrupt ischaemia and venous pressure create a strong VEGF signal that may evolve as collateral circulation develops. These spatial and temporal differences help to explain why a common labelled dose does not yield a common interval across diseases or eyes.

### 3.2. Binding Architecture and the VEGF Sink

Neutralisation depends on more than equilibrium affinity. The injected molar amount, association and dissociation kinetics, valency, accessibility of epitopes and stoichiometry of drug–ligand complex formation determine how much VEGF can be bound before free ligand reappears. Aflibercept was derived from VEGF-receptor domains fused to an Fc scaffold and binds VEGF-A, VEGF-B and PlGF [14,15]. Its receptor-trap architecture and complex stoichiometry differ from those of an antibody or Fab fragment [16]. These distinctions affect neutralising capacity and the relationship between measured total drug, free drug and free VEGF.

The eye is also a ligand sink. Newly produced VEGF can bind therapeutic molecules, while extracellular matrix and receptor surfaces buffer both ligand and drug. If the injected concentration is far above the effective threshold, several elimination half-lives can pass before control is lost. Conversely, high target abundance, rapid ligand turnover or poor access to the active compartment can shorten suppression without changing the terminal elimination slope. The most useful pharmacodynamic quantity is, therefore, not a nominal affinity value in isolation, but the duration for which unbound ligand and downstream activity remain below a prespecified biological threshold.

### 3.3. Angiopoietin–Tie2 Signalling and Pathway Interaction

The angiopoietin–Tie2 axis regulates vascular stability and modifies the response to VEGF. Angiopoietin-1 generally supports Tie2 activation, endothelial quiescence and barrier integrity. Angiopoietin-2 is context-dependent: in inflamed or VEGF-rich tissue it commonly antagonises this stabilising signal and sensitises vessels to permeability and inflammatory cues, whereas its effects on sprouting, regression and repair depend on ligand balance, Tie2 expression, integrin signalling and disease stage [17]. In diseased retina, simultaneous VEGF-A and angiopoietin-2 inhibition may, therefore, reduce leakage through complementary mechanisms rather than by simply increasing the duration of VEGF-A binding.

Faricimab embodies this pathway-expansion strategy: one arm binds VEGF-A and the other angiopoietin-2, while preserving angiopoietin-1-mediated Tie2 signalling; Fc engineering is intended to reduce systemic effector interactions [18]. Phase 2 DMO data provided clinical evidence that dual-pathway inhibition can alter anatomical response and dosing potential [19], although the relative contribution of angiopoietin-2 suppression cannot be isolated from differences in dose, format and trial algorithm. Experimental selective angiopoietin-2 blockade can spare quiescent vessels, whereas inhibition during active sprouting or ischaemic repair may constrain adaptive expansion [20]. No direct evidence currently demonstrates adverse remodelling of healthy adult human retinal or choroidal vasculature during prolonged faricimab exposure, but subtle long-term effects have not been excluded. A long clinical interval under dual inhibition should, therefore, be interpreted as stage-dependent systems-level vascular control, not as direct proof of a longer anti-VEGF half-life.

### 3.4. From Molecular Suppression to Anatomical Recurrence

Free-ligand recovery does not translate instantaneously into optical coherence tomography fluid recurrence. Receptor activation, junctional changes, fluid accumulation and detectability introduce delays, while vascular stabilisation may outlast measurable ligand suppression. The reverse is also possible: cystic spaces, degenerative tubulation or chronic structural change can persist despite adequate VEGF neutralisation. A molecular endpoint should, thus, be positioned in a causal chain—exposure, target engagement, signalling, vascular permeability, anatomy, vision—rather than treated as a substitute for the whole chain.

This biological framework makes two predictions. First, increasing dose can extend suppression even when elimination is unchanged because more half-lives are required to reach the effective threshold. Second, adding a relevant target can extend anatomical control without extending VEGF-A suppression if downstream vascular stability improves. Both predictions recur in the clinical and delivery-platform evidence considered below.

## 4. Molecular Architecture and Determinants of Ocular Persistence

### 4.1. Therapeutic Formats

Intravitreal anti-VEGF agents span several molecular formats. Bevacizumab is a full-length bivalent immunoglobulin G [21]; ranibizumab is a monovalent affinity-matured Fab fragment; aflibercept is a dimeric receptor-domain–Fc fusion protein; conbercept is a bivalent VEGFR1-D2/VEGFR2-D3-D4–Fc fusion; brolucizumab is a compact single-chain variable fragment, and faricimab is an asymmetric bispecific antibody. These architectures alter molecular mass, hydrodynamic behaviour, binding valency, target spectrum and systemic handling. Table 2 summarises the principal molecular features and approximate molar exposures delivered by commonly used doses.

Smaller constructs can deliver a larger number of molecules per injected milligram and may diffuse rapidly through the vitreous and retina, but a small size does not guarantee long residence. Brolucizumab illustrates the distinction: its compact format permits a high molar dose within the intravitreal volume [23]; however, the same hydrodynamic properties may favour faster movement towards elimination pathways. Full-length antibodies have a larger hydrodynamic radius and bivalent binding but may penetrate tissue differently and form larger complexes. Receptor traps and bispecific antibodies add further determinants that cannot be reduced to molecular mass alone.

### 4.2. Size, Charge, Diffusion and Elimination

After intravitreal injection, macromolecules distribute through a collagen–hyaluronan matrix and leave predominantly through anterior pathways, with posterior routes and tissue binding contributing according to molecular properties. Hydrodynamic radius often predicts vitreous movement better than nominal molecular mass; experimental rabbit data show a relationship between antibody size and vitreous pharmacokinetics [24]. However, species-specific ocular dimensions and retinal barriers limit direct numerical scaling to humans [25,26]. Anatomically realistic models likewise show that injection site, convection, diffusion and eye geometry can alter local concentration–time profiles [27].

Electrostatic interactions add another layer. A molecule’s charge distribution and isoelectric point can promote or limit association with vitreous components, internal limiting membrane or extracellular matrix. Retention may increase whole-eye persistence while reducing the freely diffusible fraction at the disease site. Conversely, rapid distribution may improve target access but also accelerate movement into an elimination compartment. Molecular engineering must, therefore, optimise effective target-site exposure, not maximal retention in any compartment.

### 4.3. Dose, Molar Exposure, Affinity and Stoichiometry

The injected mass dose is not a comparable measure across formats. Dividing mass by molecular mass shows that 6 mg brolucizumab delivers far more molecules than 0.5 mg ranibizumab, while aflibercept 8 mg delivers four times the molecular amount of 2 mg of aflibercept. These calculations do not account for valency or functional binding sites but show that the starting exposure can vary by an order of magnitude before affinity and distribution are considered.

Affinity affects the concentration required to maintain binding, whereas the kinetic off-rate affects the persistence of an individual complex. In vivo suppression also depends on ligand production and complex clearance. The different VEGF-binding stoichiometries reported for aflibercept and antibody-based agents illustrate why potency cannot be inferred from a single affinity constant [15,16]. A high-affinity molecule delivered at high molar exposure may remain above a functional threshold for several weeks after most of the initial dose has been eliminated.

### 4.4. Fc, Complex Handling and Compartmental Separation

Fc-containing molecules differ from Fab and single-chain fragments in size, valency and systemic handling. Experimental data suggest that the Fc region can influence vitreous persistence [28], but ocular and systemic effects should not be conflated. Once the drug leaves the eye, FcRn interactions, systemic target-mediated disposition and plasma clearance shape a different concentration–time profile. A prolonged plasma terminal slope may reflect slow release from the eye rather than persistent retinal exposure.

Assays can further blur these distinctions. Some measure free therapeutic, others total therapeutic or ligand-binding capacity; drug–ligand complexes may be recovered differently according to epitope and sample preparation. A concentration reported in aqueous humour may represent slow transfer from the vitreous rather than local elimination. Every half-life claim should therefore specify species, compartment, dose, sampling design, analyte definition and model.

### 4.5. The Time-to-Threshold Model

**Figure 2:**
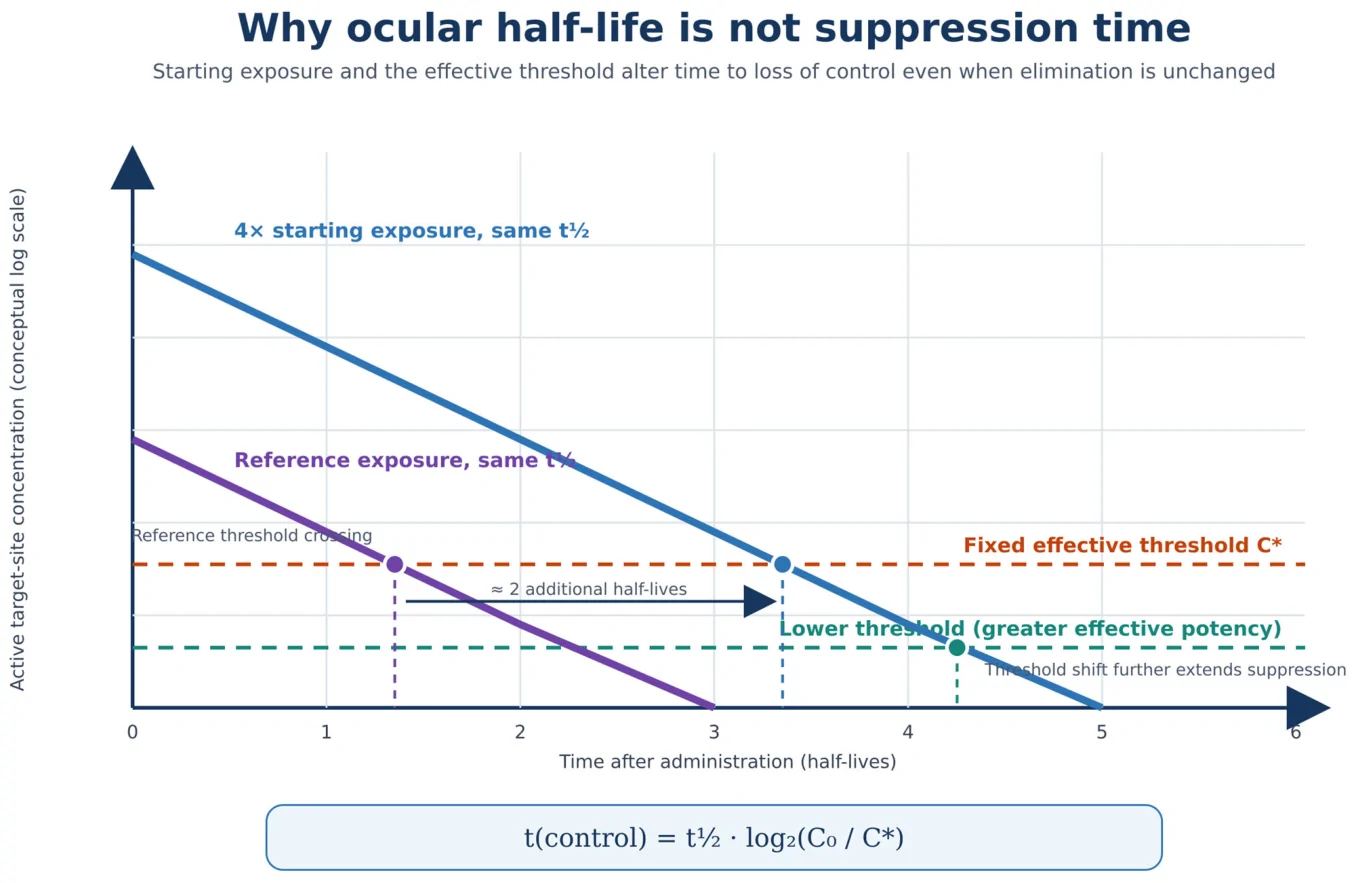
Why ocular half-life is not suppression time. For approximate first-order elimination, threshold-crossing time depends on the starting concentration (C_0_), elimination half-life (t½) and effective concentration threshold (C*). At the same half-life, a fourfold higher starting concentration adds approximately two half-lives before a fixed threshold is crossed. Higher potency or altered target biology can lower the effective threshold without changing elimination. The diagram is conceptual and not drawn from a head-to-head clinical dataset.

For approximate first-order elimination, the concentration after time t can be represented as C(t) = C_0_·2^(−t/t½)^. The suppression time for a fixed effective threshold C* is, therefore, t = t½·log_2_(C_0_/C*). This expression is deliberately simple, but it clarifies why half-life alone is insufficient. A fourfold increase in starting concentration adds approximately two half-lives before threshold crossing; a lower effective threshold produced by higher potency can have a similar effect. Figure 2 visualises these relationships.

The threshold is not universal. It can change with disease activity, target production, tissue access and the outcome chosen to define failure. A molecular threshold for detectable free VEGF, an anatomical threshold for recurrent intraretinal fluid and a protocol threshold for rescue may occur at different times. The following sections, therefore, separate ocular concentration evidence from molecular target suppression and clinical interval.

## 5. From Ocular Pharmacokinetics to Molecular Target Suppression

Ocular pharmacokinetics and pharmacodynamics are related but non-interchangeable components of durability. The ocular elimination half-life describes the rate at which a measured drug concentration declines within a specified compartment and under a specified analytical model. It does not directly indicate how long the concentration remains above a biologically effective threshold, how long free ligand remains suppressed, or when exudation recurs. The latter outcomes depend additionally on the injected molar dose, binding affinity and stoichiometry, target abundance and replenishment, tissue distribution, assay definition and the disease-specific threshold for renewed vascular leakage. Consequently, a modest difference in half-life may generate a larger difference in target-suppression time when the starting concentration or effective potency differs substantially; conversely, a longer estimated half-life need not produce a proportionately longer clinical interval. The exposure-to-threshold logic that separates half-life from suppression time is illustrated in Figure 2.

### 5.1. An Interpretive Hierarchy for Ocular PK/PD Evidence

The available literature should, therefore, be interpreted using a defined evidence hierarchy rather than treated as a single pool of comparable half-life values. At the highest level are serial drug-concentration measurements from the same human eyes in the compartment most relevant to the biological question. Such studies are rare because repeated vitreous sampling is not ethically or practically feasible. Cross-sectional human ocular studies, in which different eyes contribute one aqueous-humour sample at different times, provide direct measurements but rely more heavily on population assumptions. Human population pharmacokinetic models can integrate aqueous-humour and plasma data from larger trial datasets, but their vitreous estimates remain conditional on compartmental structure and parameter assumptions. Longitudinal human pharmacodynamic studies of free VEGF-A or Ang-2 add mechanistic information, although they measure a surrogate compartment rather than retinal or choroidal target engagement. Animal studies, interspecies scaling and purely mechanistic simulations are useful for hypothesis generation but should not be presented as equivalent to direct human ocular observations [25,26,29].

This hierarchy is interpretive rather than a formal risk-of-bias grading system. It is used here to identify the source and basis of each estimate, consistent with the narrative design of the review and its SANRA-guided critical appraisal. Four evidence classes are applied in Table 3: A, serial human ocular measurements; B, cross-sectional or longitudinal human ocular measurements; C, human population PK or PK/PD modelling; and D, preclinical or purely mechanistic modelling. A single agent may have evidence in more than one class.

Compartment and assay must be specified alongside every estimate. After intravitreal administration, an aqueous-humour terminal slope may be governed partly by slow transfer from the vitreous—the so-called flip-flop phenomenon—rather than by elimination from the anterior chamber alone. Aqueous-humour sampling is, therefore, clinically accessible and potentially informative; however, it is not identical to vitreous, retinal, subretinal, or choroidal exposure. Estimates also vary according to whether an assay detects free drug, total drug, unbound ligand, or drug–ligand complexes; whether values below the lower limit of quantification are censored or imputed; and whether one- or multi-compartment kinetics are assumed. Numerical estimates generated under different conditions should be juxtaposed, not averaged.

### 5.2. What Human Ocular Concentration Studies Show

For ranibizumab and bevacizumab, the most frequently cited human estimates derive from prospective cross-sectional studies in non-vitrectomised eyes undergoing cataract surgery. Following 0.5 mg ranibizumab, aqueous-humour concentrations from 18 eyes sampled 1–37 days after injection yielded an estimated half-life of 7.19 days [31]. A similarly designed study of 1.5 mg bevacizumab in 30 eyes sampled 1–53 days after injection estimated an aqueous-humour half-life of 9.82 days [30]. These data are direct human measurements, but each participant contributed a single postoperative sample; neither study provided serial vitreous measurements from the same eye. The numerical difference between the two agents should, therefore, be interpreted as a population-level aqueous comparison rather than proof of a fixed difference in retinal residence.

For 2 mg of aflibercept, a prospective study obtained serial aqueous-humour and plasma samples from five treatment-naïve eyes with neovascular age-related macular degeneration over 28 days. The median aqueous-humour half-life of free aflibercept was approximately 11 days [34]. This is among the most direct longitudinal human ocular PK datasets for an anti-VEGF agent; however, its sample size is too small to define the full distribution of inter-eye variability. For 8 mg of aflibercept, an independently established serial human ocular half-life is not yet available. Because the 2 mg and 8 mg products contain the same binding protein, a longer time above an effective concentration can arise from the fourfold greater dose without requiring a longer intrinsic elimination half-life. Under linear first-order kinetics, a fourfold increase in starting concentration adds approximately two half-lives before a fixed concentration threshold is crossed. Claims of materially slower ocular clearance with the 8 mg dose remain dependent on modelling assumptions and have been debated [29,37].

Faricimab illustrates the value and limitations of population modelling. A pooled analysis used 1095 aqueous-humour concentrations from 284 patients and 8372 plasma concentrations from 2246 patients across phase 1–3 studies. A sequential vitreous–aqueous–plasma model identified vitreous-to-aqueous transfer as the slowest process and estimated a 7.5-day half-life; parallel terminal slopes across compartments were attributed to flip-flop kinetics [41]. The large dataset improves precision, yet the reported vitreous value is model-derived rather than obtained by serial vitreous sampling.

Brolucizumab demonstrates why method-specific discordance must remain visible. Earlier PK simulations generally used a vitreous half-life of approximately 4.4–5.1 days, based largely on preclinical and translational assumptions [40]. In contrast, a 2025 cross-sectional human study using nSMOL liquid chromatography–tandem mass spectrometry estimated aqueous-humour half-lives of 9.00 days for brolucizumab and 2.88 days for aflibercept [39]. The study used 52 brolucizumab-treated and 59 aflibercept-treated eyes; however, most samples were obtained near week 4, the treatment groups differed in age and posterior vitreous detachment prevalence, and concentrations below quantification were imputed as one-half of the detection limit. The resulting estimates conflict with earlier size-based expectations and with the small serial aflibercept study. These are important human data but do not justify replacing one universal half-life value with another.

For conbercept, published ocular pharmacokinetic evidence remains preclinical. After a 0.5 mg intravitreal dose, the reported rabbit vitreous half-life was approximately 4.24 days [38]. No direct serial human vitreous or aqueous concentration study, and no comparable longitudinal human free-ligand suppression series, was identified. The rabbit estimate is, therefore, useful for mechanistic comparison but should not be rescaled into a human treatment interval.

### 5.3. Target Suppression as the Molecular Bridge

Target-suppression time provides a molecular bridge between exposure and disease control. In neovascular age-related macular degeneration, repeated aqueous-humour sampling before ranibizumab retreatment showed complete VEGF-A suppression for a mean of 36.4 ± 6.7 days, with a range of 26–69 days; within-eye suppression times were comparatively stable over prolonged follow-up [32]. In diabetic macular oedema, the corresponding mean after ranibizumab was 33.7 ± 5.1 days, with a range of 27–42 days [33]. These observations help explain why an ocular half-life of approximately one week can support molecular suppression for several weeks: drug concentrations begin far above the concentration required to bind measurable free VEGF-A.

Aflibercept provides a stronger example of the non-linearity between half-life and pharmacodynamic duration. In a prospective aqueous-humour study, VEGF-A remained below the assay’s lower limit of quantification for a mean of more than 71 ± 18 days after aflibercept treatment [35]. A subsequent comparative analysis reported mean VEGF-suppression times of 67 ± 14 days for aflibercept and 34 ± 5 days for ranibizumab [36]. The approximately twofold difference in molecular suppression is larger than the separation between their reported aqueous half-lives. Higher molar exposure relative to the effective threshold, high-affinity ligand trapping, binding stoichiometry, and assay-specific detection of free VEGF-A are plausible contributors. These data support target suppression as a more proximal measure of biological exposure than half-life alone, but not as a validated surrogate for retreatment interval.

Direct longitudinal human target-suppression data are less complete for brolucizumab. A mechanistic analysis predicted a mean VEGF-suppression duration of approximately 51 days for brolucizumab 6 mg and 71 days for aflibercept 2 mg, using assumed half-lives and a common suppression threshold [40]. These are conditional model outputs and not observed aqueous or retinal suppression times. They are useful for examining the implications of dose and molecular size, but they should not be used as direct comparative clinical measurements.

For faricimab, human aqueous-humour measurements from approximately 300 participants were incorporated into a population PK/PD model of free Ang-2 and VEGF-A. The model predicted at least 50% suppression from baseline for approximately 12–14 weeks for Ang-2 and 9–10 weeks for VEGF-A, depending on disease and regimen [42]. The longer predicted Ang-2 suppression is biologically relevant to the bispecific design, but it remains a model-based aqueous biomarker result. A high proportion of post-dose Ang-2 observations were below quantification; aqueous suppression cannot demonstrate the magnitude or duration of Tie2-pathway modulation within retinal tissue. The faricimab PK/PD analyses were also sponsor-supported; detailed reporting of model structure and sensitivity analyses is, therefore, particularly important.

### 5.4. Why Aqueous Ligand Suppression Is Not Retinal Target Engagement

Aqueous-humour biomarker studies allow for repeated sampling in patients, but four limitations constrain their biological interpretation. First, the anterior chamber is a surrogate compartment: aqueous VEGF-A or Ang-2 may correlate with posterior-segment activity, yet the relationship is influenced by diffusion, convection, ocular barriers, local production, and disease phenotype. Second, most assays quantify free rather than total ligand. Therapeutic binding can mask epitopes, alter recovery, or generate complexes that are not detected; a value below quantification, therefore, means ‘not measurable by this assay’, not a complete absence of ligand. Third, the end of measurable suppression is threshold-dependent. Changing the analytical lower limit or the definition of suppression can change the reported duration without changing the underlying biology. Fourth, molecular recurrence and anatomical recurrence need not be synchronous: a detectable rise in aqueous VEGF-A may precede fluid recurrence, whereas persistent structural control may reflect downstream vascular stabilisation after free ligand begins to recover.

Target suppression should, therefore, be presented as an intermediate mechanistic endpoint rather than a substitute for retinal drug concentration, receptor occupancy, optical coherence tomography control, or vision. The most convincing durability evidence is triangulated: measured ocular exposure, a prespecified molecular pharmacodynamic response, anatomical disease control, and a prospectively defined retreatment interval should move coherently within the same population.

### 5.5. Systemic Pharmacokinetics Should Be Kept Separate

Systemic exposure after intravitreal injection is relevant to systemic pharmacology and safety, but it cannot be used to infer ocular durability directly. Fc-containing agents, Fab fragments, and engineered antibodies differ in escape from the eye, systemic distribution, FcRn interactions, target-mediated disposition and plasma clearance. A longer systemic half-life may coexist with either longer or shorter ocular residence, while very low plasma concentrations can still display a terminal slope governed by slow ocular release. Comparisons of systemic VEGF suppression among bevacizumab, aflibercept, ranibizumab and faricimab should, therefore, remain analytically separate from estimates of intravitreal residence and retinal target control [41,43,44].

### 5.6. Synthesis of Ocular PK/PD Evidence

The available human evidence supports three conclusions. First, reported ocular half-lives are method- and compartment-specific parameters rather than immutable molecular constants. Second, the duration of measurable target suppression can diverge substantially from half-life because it integrates dose, potency, binding stoichiometry, target turnover, and analytical threshold. Third, neither aqueous concentration nor aqueous ligand suppression alone establishes retinal target engagement or clinical durability. Future comparative studies should prespecify the sampled compartment, distinguish free from total analyte, report handling of below-quantification values, validate the PK/PD model against observed data and relate molecular endpoints prospectively to anatomical recurrence and retreatment. This sequence of evidence is needed before molecular engineering can support a credible claim of treatment durability.

## 6. Translating Molecular Suppression into Anatomical and Clinical Durability

Clinical durability is the final expression of several upstream processes, but it is not a direct read-out of any single molecular property. A treatment interval becomes clinically credible only when disease control is maintained across the interval with acceptable functional, anatomical and safety outcomes. The chain of inference is, therefore, sequential: molecular design determines ocular exposure and target suppression; target suppression influences vascular permeability and neovascular activity; these effects shape optical coherence tomography findings and haemorrhagic activity; and protocol rules translate those observations into a retreatment interval. Each transition introduces biological variability and measurement error. Accordingly, a 12- or 16-week interval in a phase 3 trial should be described as protocol-validated clinical durability, not as proof that the molecule remains present or fully suppressive for the entire interval. Table 4 summarises how the principal molecular strategies have translated into trial-based dosing intervals and where mechanistic attribution remains uncertain. Table 5 consolidates selected efficacy, anatomical, interval, inflammatory-safety and economic evidence.

### 6.1. Protocol-Enabled Durability Is Not Intrinsic Durability

The longest interval permitted by a trial is partly a property of trial design. Treatment initiation/loading-dose number, the timing of disease-activity assessments, criteria for interval shortening or extension, tolerance of residual subretinal fluid, rescue rules and visit frequency all affect the observed distribution of intervals. Fixed dosing demonstrates that a regimen can maintain group-level efficacy at a prespecified interval; it does not identify each eye’s biological ceiling. Conversely, treat-and-extend studies estimate an interval distribution, but that distribution is conditional on the algorithm used. A trial in which an interval can only shorten will generate a different durability estimate from one that permits re-extension, even if the underlying drug and patient population are identical.

Non-inferiority also requires careful interpretation. Similar mean best-corrected visual acuity does not establish equivalent molecular suppression, identical fluid control, or the same proportion of patients remaining on the assigned interval. Visual acuity remains a patient-centred endpoint but is relatively insensitive to some differences in retinal fluid, pigment epithelial detachment, haemorrhage or disease fluctuation. Therefore, the most informative clinical appraisal considers the assigned and achieved interval, the interval-modification rules, the proportion requiring rescue or shortening, anatomical control, injection number and safety together rather than in isolation.

### 6.2. The First Clinical Benchmark: Sustained VEGF Suppression

Monthly ranibizumab established that continuous VEGF-A inhibition could improve vision in neovascular age-related macular degeneration [1]. The VIEW programme subsequently showed that 2 mg of aflibercept every 8 weeks after three initial monthly doses could maintain visual outcomes comparable with monthly ranibizumab during the fixed-dosing phase, supporting a broader-ligand, high-affinity trap as a clinically durable regimen [45]. This was an important reduction in treatment frequency, but the result cannot be attributed to half-life alone. Aflibercept differs from ranibizumab in injected molar exposure, affinity, stoichiometry and target spectrum; its measured aqueous VEGF-suppression time is substantially longer [35,36]. The VIEW regimen, thus, provides clinical consistency with the molecular pharmacodynamic evidence, without proving that aqueous suppression caused the 8-week efficacy interval.

The same principle applies to bevacizumab and ranibizumab. Monthly, pro re nata and treat-and-extend regimens have produced different injection frequencies with the same molecule, demonstrating that clinical durability is co-determined by the monitoring and retreatment strategy. Regimen comparisons should not be used to assign a unique clinical half-life to an agent.

### 6.3. Receptor-Domain Engineering: Conbercept

Conbercept is an approximately 143-kDa bivalent VEGF-receptor–Fc fusion comprising VEGFR1 domain 2 and VEGFR2 domains 3 and 4 linked to human IgG1 Fc. Direct binding studies demonstrate recognition of VEGF-A isoforms, VEGF-B and PlGF, although absolute affinity estimates are assay-dependent [22]. At the standard 0.5 mg dose, approximately 3.5 nmol of fusion protein is delivered. Rabbit vitreous pharmacokinetics do not substitute for a direct human half-life estimate [38].

AURORA evaluated 0.5 and 2.0 mg doses under monthly or pro re nata schedules [59]. In the China-based PHOENIX trial, three monthly 0.5 mg injections followed by dosing every 12 weeks produced a mean gain of 9.20 letters and a central retinal-thickness reduction of 125.92 μm at month 3 versus 2.02 letters and 40.67 μm with sham; after delayed crossover, the corresponding month-12 visual gains were 9.98 and 8.81 letters [46]. These findings support activity and maintenance within that protocol, but PHOENIX did not include a contemporary active anti-VEGF comparator. The multinational PANDA-1 and PANDA-2 comparisons with aflibercept were terminated because the desired primary endpoint was not met [47,48]. Current evidence, therefore, identifies conbercept as an informative receptor-domain design without establishing superior durability to active comparators.

### 6.4. High Molar Payload Within a Small Scaffold: Brolucizumab

Brolucizumab was designed to exploit a small single-chain variable fragment to deliver a high molar dose of VEGF-A-binding sites in a 0.05 mL injection. In HAWK and HARRIER, eyes received brolucizumab every 12 weeks after treatment initiation (loading), with permanent adjustment to every 8 weeks when prespecified disease activity was detected; 2 mg of aflibercept was administered every 8 weeks. At the final disease-activity assessment near week 92, the probabilities of maintaining the 12-week regimen with 6 mg of brolucizumab were 45.4% in HAWK and 38.6% in HARRIER. Mean vision gains were similar to aflibercept, while brolucizumab produced greater central subfield thickness reductions and lower proportions of eyes with intraretinal or subretinal fluid [49]. These results show that high molar loading can support extended control in a substantial subset, but not that every eye is biologically controlled for 12 weeks.

Brolucizumab also demonstrates that the practical value of an extended interval must be interpreted as safety-adjusted durability. An independent post hoc review of HAWK and HARRIER reported definite or probable drug-related intraocular inflammation in 4.6% of brolucizumab-treated eyes, with vasculitis in 3.3% and vasculitis with vascular occlusion in 2.1%; at least moderate vision loss occurred in a smaller subset [50]. Translational analyses support a multifactorial, product-specific immune mechanism involving anti-drug responses, a sequence epitope, exposed surfaces of this construct, non-native drug species that may arise during intraocular residence, dose intensity and host susceptibility [51]. These findings neither reduce the event to manufacturing aggregates nor establish that the scFv class is intrinsically unsafe. Aggregate-resistant design, optimised refolding and purification, and orthogonal control of aggregates, particles and host-cell impurities remain rational strategies for reducing immunogenicity risk [51]. Whether these measures reduce the clinical incidence of brolucizumab-associated retinal vasculitis or vascular occlusion remains uncertain [50,51].

### 6.5. Pathway Diversification: Faricimab

Faricimab tests a different hypothesis: that simultaneous inhibition of VEGF-A and Ang-2 can extend disease control by combining suppression of permeability and angiogenesis with stabilisation of the Tie2 vascular pathway. In TENAYA and LUCERNE, faricimab administered at intervals of up to 16 weeks produced non-inferior visual outcomes to 2 mg of aflibercept every 8 weeks through the first year [52]. During year 2, a protocol-driven treat-and-extend regimen allowed for adjustment between 8 and 16 weeks; vision and anatomical improvements were maintained, with most faricimab-treated patients achieving 12- or 16-week dosing [53]. In YOSEMITE and RHINE, faricimab treat-and-extend similarly maintained visual gains and anatomical control in diabetic macular oedema, with more than 60% of patients receiving 16-week dosing and approximately 80% receiving 12-week or longer dosing at week 96 [54].

The faricimab PK/PD data make dual-pathway durability biologically plausible: modelled aqueous Ang-2 suppression persisted longer than VEGF-A suppression [42]. However, the trials did not randomise patients to Ang-2 inhibition alone versus VEGF-A inhibition alone, and the population PK/PD analyses do not establish that an individual patient’s Ang-2 suppression duration mediates that patient’s achieved treatment interval. The clinical evidence validates the faricimab regimen; attribution of the full interval benefit specifically to Ang-2 remains a mechanistic inference. The distinction is important because faricimab also delivers a high molar dose, has engineered Fc properties, and was tested with a disease-activity algorithm different from the fixed aflibercept comparator. The biological effect of Ang-2 suppression may differ between acute ischaemic sprouting and chronic stabilisation; long-term retinal and choroidal microvascular surveillance remains appropriate [17,20].

### 6.6. Dose Intensification Without a New Target: Aflibercept 8 mg

Aflibercept 8 mg isolates dose intensification more cleanly because the binding protein and ligand spectrum are unchanged relative to aflibercept 2 mg. PULSAR in neovascular age-related macular degeneration and PHOTON in diabetic macular oedema evaluated 8 mg every 12 or 16 weeks after initial monthly dosing against 2 mg of aflibercept every 8 weeks. At week 48, the extended 8 mg regimens were non-inferior for visual acuity. In PULSAR, 79% of patients assigned to every-12-week dosing and 77% assigned to every-16-week dosing maintained their original intervals; the corresponding proportions in PHOTON were 91% and 89% [55,56]. Thus, increasing the initial concentration can extend the time above an effective threshold even without changing the molecule’s intrinsic elimination rate.

The 96-week PULSAR and PHOTON reports showed sustained visual and anatomical outcomes with fewer injections, and year-2 algorithms permitted selected eyes to extend as far as 24 weeks [57,58]. These longer intervals are strong evidence for clinical dose-dependent durability. They do not, however, demonstrate that 8 mg of aflibercept has a distinct ocular half-life from 2 mg of aflibercept. The most parsimonious molecular explanation remains prolonged exposure above a pharmacologically effective concentration, potentially augmented by formulation-dependent distribution, rather than a fourfold change in clearance [29,37].

### 6.7. Anatomical Control and Visual Function Are Related but Non-Equivalent

Anatomical durability is generally more sensitive than visual acuity to incomplete molecular control. Intraretinal fluid, subretinal fluid, retinal thickness and haemorrhage can recur before a statistically detectable decline in mean vision. Conversely, an eye may retain stable visual acuity despite residual or fluctuating fluid because photoreceptor reserve, lesion location and chronic structural damage modify the functional response. Greater drying in one treatment group should, therefore, not automatically be translated into superior functional durability, while non-inferior vision should not be taken to mean identical biological suppression.

Disease-activity definitions further influence the apparent interval. Intraretinal fluid is usually treated as a strong signal for retreatment, whereas small, stable quantities of subretinal fluid may be tolerated in some neovascular age-related macular degeneration algorithms. Diabetic macular oedema adds systemic and inflammatory determinants of leakage that may dissociate central thickness from VEGF-A concentration. A molecularly focused review should, therefore, report which of the anatomical features triggered interval modification rather than reducing durability to the absence of any fluid.

### 6.8. Transferability to Routine Practice

Trial durability is an efficacy estimate under structured monitoring. Routine clinical populations include previously treated eyes, incomplete treatment responders, fibrosis, macular atrophy, vitreomacular interface abnormalities, variable adherence and longer delays between assessment and treatment. Switching studies are especially difficult to interpret mechanistically because eyes are selected for inadequate response or treatment burden; baseline intervals reflect the preceding drug and clinic policy; and loading after a switch may create a transient anatomical improvement. Regression to the mean can also mimic a molecular benefit. Real-world interval extension is clinically important; however, causal attribution requires adjustment for previous treatment intensity, disease phenotype, loading strategy and follow-up opportunity.

The most defensible real-world endpoints are the paired change in achieved interval; proportion remaining dry or clinically stable at that interval; annualised injection and visit numbers; interval-shortening rate; and discontinuation for inefficacy or adverse events. Reporting only the final mean interval risks survivor bias because patients who discontinue or switch again are removed from the durability denominator.

### 6.9. Safety, Treatment Burden and Economic Context

Repeated intravitreal therapy imposes risks that are partly procedure-related and partly molecule- or product-specific. Transient intraocular-pressure elevation, discomfort and subconjunctival haemorrhage are common, whereas endophthalmitis, retinal tear or detachment, lens injury, vitreous haemorrhage and severe sterile inflammation are uncommon but potentially sight threatening [60]. Cumulative injections also entail anxiety, transport and caregiver requirements, and consequences from treatment gaps. These risks should be distinguished from the persistent device-, depot- or transgene-related risks considered in Section 8.

Intravitreal anti-VEGF proteins can reach the systemic circulation and differ in systemic exposure and free-VEGF suppression. A meta-analysis of randomised trials found no clear overall increase in major cardiovascular events, but uncommon events and signals in high-risk subgroups remain difficult to exclude [61]. Clinical decisions should, therefore, account for recent systemic vascular events, pregnancy and other patient-specific factors without inferring systemic risk from ocular half-life alone.

Acquisition cost is not an intrinsic molecular property and should not be conflated with pharmacological durability. Economic value varies with jurisdiction, negotiated price, biosimilar availability, compounding arrangements, treatment frequency and the costs of monitoring, administration, travel, caregiver time, rescue and adverse events. Trial-based analyses in nAMD and DMO have demonstrated substantial savings with appropriately repackaged off-label bevacizumab relative to branded agents under specific UK and US assumptions [62,63]. Chinese modelling has suggested that conbercept may be cost-effective within that health system, but the conclusion is price year- and jurisdiction-specific [64]. Extended-interval regimens may offset higher acquisition costs through fewer injections; however, estimates for brolucizumab, faricimab and 8 mg of aflibercept depend strongly on achieved interval, safety assumptions and local prices. Economic evidence should, therefore, be reported by indication, perspective and price year rather than as a universal ranking.

### 6.10. Synthesis of Clinical Durability Evidence

The clinical trial programme supports several molecular routes to longer anti-VEGF treatment intervals: receptor-domain engineering; higher molar loading within a compact scaffold; pathway diversification beyond VEGF-A; and a higher dose of an established ligand trap. None can be ranked by the nominal maximum interval alone. A credible comparison requires harmonised loading, interval-adjustment rules, disease-activity thresholds, anatomical definitions, follow-up duration, safety and cost perspective. Randomised trials establish that particular regimens can maintain clinical outcomes; they do not by themselves reveal which molecular step generated the interval. The strongest causal claim arises when exposure, target suppression, anatomical control and retreatment interval are measured prospectively in the same eyes. Until such data are available, clinical durability should be described as molecularly supported but protocol-validated.

**Table 5:** Selected efficacy, anatomical, interval, inflammatory-safety and economic evidence from pivotal nAMD programmes.

Agent and Pivotal Programme	Visual-Acuity Outcome	Anatomical Outcome	Protocol Interval	Inflammatory/Procedural Safety	Economic and Access Interpretation
Bevacizumab 1.25 mg (off-label) CATT [65]	Month 12: +8.0 letters monthly and +5.9 as needed; corresponding ranibizumab gains were +8.5 and +6.8.	Mean total-foveal-thickness change: −164 μm monthly and −152 μm as needed; monthly ranibizumab −196 μm.	q4w or as needed with monthly assessment; mean 11.9 and 7.7 injections in year 1.	Endophthalmitis: 0.07% of injections versus 0.04% with ranibizumab; uveitis and other serious ocular events each occurred in <1%.	Usually the lowest acquisition-cost option when safely compounded; IVAN found branded ranibizumab unlikely to be cost effective versus bevacizumab at contemporaneous UK prices, but access and compounding governance vary [62].
Ranibizumab 0.5 mg MARINA [1]	+7.2 ETDRS letters at month 12.	No directly comparable modern CST endpoint in the pivotal report.	q4w; a longer interval was not tested.	Across ranibizumab groups over 24 months: presumed endophthalmitis 1.0%; serious uveitis 1.3%.	Branded efficacy benchmark; substantially more costly than safely compounded bevacizumab in older UK analyses; biosimilars and contracts may narrow the difference [62].
Aflibercept 2 mg VIEW [3,45]	+8.4 letters at week 52 with 2q8; monthly ranibizumab +8.7.	Approximately 130 μm mean reduction across randomised arms at week 52; arm-specific definitions and pooling limit direct comparison.	q8w after three monthly doses; year-2 dosing at least q12w with additional treatment as needed.	IOI 0.5–1.1% across aflibercept arms through 96 weeks versus 1.5% with ranibizumab.	Fewer injections than monthly ranibizumab; US DMO models favour bevacizumab-first/switch at prevailing prices, but value depends on indication and baseline vision [63].
Conbercept 0.5 mg PHOENIX [46]	Month 3: +9.20 letters versus +2.02 with sham; month 12 after crossover: +9.98 versus +8.81.	Month 3 CRT: −125.92 μm versus −40.67 μm with sham.	q12w after three monthly doses.	Common ocular events were injection related; *n* = 124 and the delayed-treatment design cannot quantify rare product-specific inflammation.	A Chinese model suggested cost effectiveness under 2018 local prices; the conclusion is not transferable across health systems [64].
Brolucizumab 6 mg HAWK/HARRIER [49,50]	Week 96: +5.9 and +6.1 letters.	Week 96 CST: −174.8 and −197.7 μm.	Probability of maintaining q12w at week 92: 45.4% and 38.6%; otherwise permanently q8w.	Adjudicated drug-related IOI 4.6%; retinal vasculitis 3.3%; vasculitis with occlusion 2.1%.	Fewer injections are possible for a subset, but inflammation, monitoring and discontinuation can reduce safety-adjusted and economic value.
Faricimab 6 mg TENAYA/LUCERNE [52]	Average over weeks 40, 44 and 48: +5.8 and +6.6 letters.	Average CST change: −136.8 and −137.1 μm.	q16w: 45.7% and 44.9%; at least q12w: 79.7% and 77.8% at week 48.	IOI 1.5% and 2.4% through week 48; no retinal vasculitis or occlusive vasculitis reported.	Potential administration savings depend on the achieved interval, acquisition price and local monitoring pathway; direct generalisable comparisons remain limited.
Aflibercept 8 mg PULSAR [55,57]	Week 48: +6.7 (q12w) and +6.2 (q16w) versus +7.6 with aflibercept 2 mg q8w.	Week 48 CST: −142 and −147 μm versus −136 μm with aflibercept 2 mg.	Maintained assigned q12w and q16w intervals: 79% and 77% at week 48.	Through week 96: IOI 1.3% with pooled 8 mg versus 2.1% with 2 mg.	Fewer injections may offset acquisition and administration costs; comparative value versus other long-interval agents is price-, protocol- and model-dependent.

Values are descriptive and are not valid head-to-head rankings. Trials differed in population, loading, visit schedule, anatomical definitions, interval algorithms, safety adjudication and follow-up; the reported inflammation periods also differ. Economic conclusions depend on jurisdiction, price year, negotiated discounts, biosimilar or compounding access, perspective and included costs. CATT, Comparison of AMD Treatments Trials; CRT, central retinal thickness; CST, central subfield thickness; ETDRS, Early Treatment Diabetic Retinopathy Study; IOI, intraocular inflammation; nAMD, neovascular age-related macular degeneration; q4w/q8w/q12w/q16w, every 4/8/12/16 weeks.

## 7. Patient- and Disease-Specific Modifiers of Anti-VEGF Durability

The same anti-VEGF molecule, dose and regimen can produce markedly different treatment intervals in different eyes. This heterogeneity does not invalidate molecular pharmacokinetics; it reflects the fact that clinical durability is generated at the intersection of drug exposure and disease drive. Exposure-side modifiers determine how rapidly free drug declines within the relevant ocular compartment. Target-side modifiers determine how quickly newly produced VEGF and parallel permeability signals exhaust the remaining neutralising capacity. Tissue-side modifiers determine whether a given amount of signalling produces visible fluid, haemorrhage or visual change. The observed interval ends when this combined biological state crosses the retreatment threshold selected by the clinician or protocol. Patient-specific durability is, therefore, better conceptualised as a time-to-threshold phenotype than as a fixed property of the injected molecule. The major patient- and disease-specific modifiers of observed durability are summarised in Table 6.

### 7.1. Ocular Anatomy and Surgical Status: Modifiers of Exposure

Intravitreal biologics are distributed within a dynamic ocular compartment rather than a uniform reservoir. Vitreous liquefaction, posterior vitreous separation, convection, axial length and the integrity of anterior and posterior elimination pathways can affect the spatial and temporal concentration profile. Pars plana vitrectomy is the most clinically recognisable perturbation. Removal of the formed vitreous may increase mixing and access to anterior clearance routes; however, the magnitude of this effect depends on molecular size, lens status, species and the pharmacokinetic model used.

In macaque eyes, vitrectomy shortened the duration for which aqueous VEGF remained below the assay limit after ranibizumab and aflibercept; ranibizumab suppression was reported for approximately 1 week in vitrectomised eyes versus 3 weeks in non-vitrectomised eyes, while aflibercept suppression was also shorter after vitrectomy [66]. These observations support a plausible exposure penalty, but they should not be converted into a universal human dose-adjustment rule. Human pharmacokinetic data are sparse and animal results are not fully concordant; vitrectomy may simultaneously alter retinal oxygenation and the biological drivers of diabetic macular oedema. A shorter clinical interval in a vitrectomised eye may, therefore, reflect altered clearance, altered disease biology or both.

The effects of age-related vitreous liquefaction, posterior vitreous detachment, pseudophakia and axial length are even less certain. Each may influence dilution or transport, yet none is currently a validated stand-alone predictor of the anti-VEGF interval. These factors are best treated as covariates in mechanistic studies rather than reasons to prescribe a different interval before the eye’s response has been observed.

### 7.2. Baseline VEGF Production and the Individual Suppression Clock

A fixed injected dose can remain pharmacologically adequate for different lengths of time because pathogenic ligand is continuously produced. An eye with a high VEGF production rate or a large accessible ligand pool consumes neutralising capacity more rapidly and reaches a biologically active free-VEGF concentration sooner. Conversely, an eye with lower target production may remain controlled after the drug concentration has fallen substantially. This target-mediated component explains why elimination half-life and suppression time are related but non-equivalent.

**Table 6:** Patient- and disease-specific modifiers that can alter the observed durability of intravitreal anti-VEGF therapy.

Modifier	Mechanistic Route	Potential Clinical Signal	Current Evidential Interpretation	Implication for Durability Assessment
Vitrectomy and vitreous state	Altered mixing, distribution and access to ocular clearance routes.	Earlier recurrence or shorter target suppression in some eyes.	Supportive animal PK/PD evidence; limited and non-uniform human evidence [66].	Record surgical and lens status; do not apply a universal interval reduction.
VEGF production and recovery	Faster ligand generation consumes binding capacity and advances threshold crossing.	Reproducible eye-specific recurrence or aqueous VEGF recovery pattern.	Moderate mechanistic clinical evidence from serial aqueous studies [32,36].	Distinguish high target demand from rapid drug clearance.
nAMD lesion and fluid phenotype	Lesion compartment and barrier integrity shape the anatomical expression of signalling.	IRF, SRF, PED or haemorrhage recur at different thresholds.	Strong prognostic evidence; molecular causality is less certain [67,68].	Report fluid compartment and the algorithm’s fluid tolerance.
Inflammatory and Ang/Tie2 activity	Non-VEGF permeability and endothelial-destabilising signals persist despite VEGF blockade.	Residual or recurrent oedema with measurable cytokine activity.	Biologically plausible; small, heterogeneous biomarker cohorts [69,70,71,72].	Consider pathway mismatch; biomarkers require prospective validation.
Retinal non-perfusion	Hypoxia sustains VEGF and inflammatory cytokine production.	High or changing treatment demand in retinal vein occlusion.	Moderate pathophysiological evidence; limited direct interval prediction [73].	Interpret short intervals as possible high demand, not necessarily rapid clearance.
Chronic structural damage	Fibrosis, atrophy and photoreceptor loss uncouple anatomy from visual function.	Stable cysts or limited visual gain despite reduced leakage.	Strong clinical association; not evidence of molecular escape by itself.	Use longitudinal change and multimodal imaging rather than a binary dry endpoint.
Systemic metabolic milieu	Hyperglycaemia, hypertension, renal disease and inflammation modify vascular permeability.	Variable oedema persistence, particularly in DMO and RVO.	Associations are inconsistent and insufficient for interval prescription [74].	Optimise systemic health, but individualise ocular dosing from observed response.
Genetics and treatment history	Inherited inflammatory/angiogenic tone or acquired compensatory responses may alter demand.	Variable injection number, incomplete response or apparent tachyphylaxis.	Conflicting pharmacogenetic evidence; tachyphylaxis evidence is mainly observational [75,76].	Do not use current genotypes or uncontrolled switching responses as causal proof.

Evidence strength refers to support for the modifier, not to a validated rule for prescribing an interval. Ang, angiopoietin; DMO, diabetic macular oedema; IRF, intraretinal fluid; nAMD, neovascular age-related macular degeneration; PED, pigment epithelial detachment; PK/PD, pharmacokinetics/pharmacodynamics; RVO, retinal vein occlusion; SRF, subretinal fluid; VEGF, vascular endothelial growth factor.

Serial aqueous humour sampling has provided clinical evidence for an individual suppression clock. During repeated ranibizumab treatment, VEGF suppression time showed substantial between-eye variation but relative intra-individual stability over extended follow-up [32]. In a separate clinical comparison, the longer aqueous VEGF-suppression time observed with aflibercept than with ranibizumab was associated with a longer interval to morphological recurrence [36]. These findings suggest that drug-specific potency interacts with an eye-specific VEGF recovery phenotype.

Important limitations remain. Aqueous humour is a surrogate compartment; concentrations near the macula may differ because of retinal production, tissue binding, diffusion across the vitreous and transport across the retinal pigment epithelium. Values below an assay’s lower limit of quantification do not establish complete receptor silence, and a recurrence detected at the next scheduled visit is only an interval-censored estimate of biological reactivation. The suppression clock is, therefore, a useful mechanistic construct, not a directly measurable clinical half-life.

### 7.3. Neovascular AMD Phenotype and the Anatomical Expression of Reactivation

Neovascular age-related macular degeneration encompasses biologically distinct macular neovascularisation phenotypes. Type 1 lesions beneath the retinal pigment epithelium, type 2 lesions in the subretinal space, type 3 lesions arising within the retina and polypoidal choroidal vasculopathy differ in vascular architecture, tissue compartment, haemorrhagic tendency and associated pigment epithelial detachment. Lesion size, maturation, fibrosis and the integrity of the outer blood–retinal barrier further influence how renewed signalling appears on optical coherence tomography. These features can change the clinical interval even when ocular drug exposure is identical.

Fluid compartment is particularly important. The Comparison of AMD Treatments Trials (CATT) analyses demonstrated that intraretinal fluid, subretinal fluid and pigment epithelial detachment have different associations with visual acuity and treatment response [67]. Intraretinal fluid usually prompts retreatment because it is strongly associated with impaired retinal function, whereas small, stable quantities of subretinal fluid may be compatible with maintained vision. In the FLUID trial, a treat-and-extend strategy that tolerated limited subfoveal subretinal fluid achieved visual outcomes non-inferior to a strategy requiring complete fluid resolution and permitted fewer injections [68]. Thus, the same biological state can generate a different reported interval depending on which fluid compartment is accepted.

Structural chronicity further separates anatomical from functional durability. Fibrosis, photoreceptor loss, disorganisation of retinal inner layers and macular atrophy can cap visual recovery even when exudation is controlled. Conversely, a stable pigment epithelial detachment or degenerative intraretinal cyst may persist despite adequate VEGF suppression. Treating every residual structural abnormality as active molecular escape risks unnecessary interval shortening; ignoring new fluid or haemorrhage risks undertreatment. Longitudinal change is generally more informative than a single, binary dry-versus-wet classification.

### 7.4. Diabetic Macular Oedema and Retinal Vein Occlusion: Multisignal Leakage

Diabetic macular oedema is not solely a high-VEGF state. Chronic hyperglycaemia, oxidative stress, leukostasis, endothelial injury and Müller-cell dysfunction generate a network that includes VEGF-A, placental growth factor, Ang-2, intercellular adhesion molecule-1, interleukins and monocyte chemoattractant protein-1. Aqueous studies have associated cytokine patterns with anatomical response, while refractory diabetic macular oedema has been linked to higher IL-8 concentrations in a small clinical cohort [70,71]. These observations provide a molecular explanation for persistent leakage despite apparently adequate VEGF neutralisation; however, the reported markers and effect directions have not been sufficiently consistent for routine interval selection.

Persistent oedema should not also be equated automatically with treatment failure. In a secondary analysis of Protocol T, persistent central thickening at 24 weeks was common, particularly with bevacizumab; however, many eyes continued to gain vision and few experienced substantial visual loss through 2 years [77]. The anatomical threshold for extension should, therefore, account for trajectory, visual function and chronic structural change rather than demanding immediate normalisation of central thickness.

In retinal vein occlusion, the continuing stimulus is closely coupled to retinal non-perfusion and hypoxia. Vitreous VEGF and IL-6 concentrations have correlated with the extent of non-perfusion and the severity of macular oedema in branch retinal vein occlusion [73]. A high or changing ischaemic burden can, consequently, shorten the interval without implying unusually rapid drug clearance. Collateral development, reperfusion and inflammatory activity may also change the demand state over time, making durability in retinal vein occlusion intrinsically dynamic rather than a fixed baseline trait.

### 7.5. Aqueous Humour Biomarkers: Promise and Measurement Constraints

Aqueous humour is attractive for precision treatment because it can be sampled at the time of an intravitreal injection. Multi-analyte models in neovascular age-related macular degeneration have combined baseline and on-treatment proteins to predict anatomical outcomes more effectively than single markers in small cohorts [69]. In diabetic macular oedema, longitudinal cytokine analysis has linked specific inflammatory mediators to higher-order optical coherence tomography features, suggesting that soluble biology and imaging phenotype can be integrated [70]. Dynamic measurements may be more informative than baseline concentrations alone because the change after treatment distinguishes target engagement from constitutively high expression.

Translation is limited by compartment mismatch, small sample sizes, multiple testing, platform-dependent assay behaviour and sampling confounders. A 2024 proteomic and metabolomic study showed that total aqueous protein concentration, pseudophakia and pharmacological pupil dilation materially altered measured protein profiles; phenylephrine/tropicamide exposure was associated with marked increases in several proteins, including IL-6 [72]. Pre-analytical standardisation, adjustment for total protein and ocular covariates, external validation and prospective testing against achieved interval are, therefore, prerequisites before an aqueous biomarker can be considered a clinical durability test.

### 7.6. Systemic and Genetic Modifiers

Systemic vascular biology can modify ocular disease drive, especially in diabetic macular oedema and retinal vein occlusion. Glycaemic control, blood pressure, renal dysfunction, dyslipidaemia and systemic inflammation are biologically plausible modifiers of endothelial permeability and VEGF expression. Some observational studies have associated lower glycated haemoglobin with better anatomical or visual response to VEGF inhibition [74], whereas others have found weak or inconsistent effects after adjustment. These variables remain important to general health and retinopathy management, but none provides a sufficiently precise rule for selecting an intravitreal interval.

Pharmacogenetics has produced a similar pattern of plausibility without clinical readiness. Variants in CFH, ARMS2/HTRA1, C3 and VEGF-pathway genes could alter complement activity, inflammatory tone or VEGF signalling. However, the CATT pharmacogenetic analysis found no significant association between major AMD risk genotypes and visual outcome, anatomical response or injection number [75], while smaller studies have reported discordant associations. Current evidence does not justify using an AMD risk genotype to choose an anti-VEGF molecule or predict durability. Future studies will require adequately powered multi-omic models, ancestry-aware validation and a prespecified interval endpoint rather than retrospective responder categories.

### 7.7. Treatment History, Apparent Tachyphylaxis and Switching

An interval that shortens after repeated injections is often labelled tachyphylaxis, but several mechanisms can produce the same clinical pattern. True pharmacodynamic attenuation could involve compensatory angiogenic pathways, receptor adaptation or an anti-drug immune response. Alternatively, lesion growth, increasing fibrosis, a change in fluid tolerance, missed visits or regression to the mean can create apparent loss of effect. A small retrospective series described loss of the previous anatomical response to bevacizumab in six eyes and found that doubling the dose did not restore it [76]. This observation established clinical plausibility but did not identify a molecular mechanism.

Switching to a molecule with a different molar dose, affinity, ligand spectrum or formulation can improve anatomy and sometimes extend the interval. The response does not prove immune tachyphylaxis to the original agent because reloading, greater VEGF-binding capacity, regression to the mean and altered protocol thresholds are alternative explanations. Mechanistic switching studies should measure anti-drug antibodies, free and total ligand, exposure, optical coherence tomography change and the subsequent interval in the same eyes. Without these data, ‘incomplete or suboptimal response’ is a safer term than molecular resistance.

### 7.8. From Population Averages to Individualised Durability

No baseline feature currently predicts a safe, long interval with sufficient accuracy to replace observed response. The most informative clinical biomarker is often the eye’s own early trajectory: speed of drying after treatment initiation/loading, completeness and compartment of fluid resolution, time to first recurrence, reproducibility of recurrence and stability after interval extension. These repeated observations implicitly integrate clearance, target production, vascular phenotype and tissue reserve. A short first interval should not be considered permanent, because disease drive may fall after vascular maturation; equally, an initially dry eye may reactivate when the interval exceeds its suppression threshold.

A precision-durability study should prospectively combine a known molecular dose with serial ocular exposure or target-suppression measurements, standardised optical coherence tomography feature quantification and protocolised interval extension. The primary endpoint should be the achieved interval without new intraretinal fluid, clinically important subretinal fluid, haemorrhage or visual loss. Models should report calibration and external validation, not only discrimination. Such a design would distinguish an eye that clears drug rapidly from one that generates ligand rapidly and from one whose leakage is maintained by non-VEGF pathways—three phenotypes that can look identical in routine optical coherence tomography but may require different therapeutic solutions.

A practical implementation would combine a hierarchical population PK/PD model with a joint longitudinal–time-to-event model. The PK/PD layer would encode agent, molar dose, binding properties and ocular covariates; molecular mass is constant within an agent and is better represented as a drug-specific prior than as a patient-level predictor. The longitudinal layer would link baseline aqueous VEGF, quantitative intraretinal-fluid, subretinal-fluid and pigment epithelial-detachment volumes, lesion phenotype and early post-loading OCT response to the interval-censored hazard of renewed activity. Penalised Cox or accelerated failure-time models provide interpretable benchmarks; gradient-boosted survival trees and random survival forests can capture nonlinear interactions, whereas deep multimodal models require large multicentre datasets. Prediction should be dynamic and probabilistic—for example, the probability of remaining below a prespecified activity threshold at 8, 12 or 16 weeks—and should be recalibrated after each injection. Baseline aqueous VEGF remains exploratory because early OCT response has been more informative than baseline cytokine concentration in distinguishing subsequent treatment demand [78]. Validation must be separated by patient and site, address scanner domain shift and label leakage, and should report calibration, Brier score and clinical decision curves in addition to discrimination.

### 7.9. Synthesis of Patient-Specific Modifiers

Patient-level anti-VEGF durability is the product of an exposure–demand–tissue interaction. Vitrectomy and ocular anatomy may modify exposure; VEGF production and parallel cytokine pathways modify demand; lesion compartment and retinal structural reserve modify the anatomical and functional expression of recurrent signalling. The current evidence is strongest for serial, eye-specific response as a predictor and weaker for single-baseline molecular, systemic or genetic markers. The practical implication is not that molecular design becomes secondary, but that its benefit must be tested across biological strata. A durable molecule raises the population efficacy and durability ceiling; patient and disease biology determine where an individual eye sits beneath that ceiling.

## 8. Emerging Molecular and Drug-Delivery Strategies for Extending Durability

**Figure 3:**
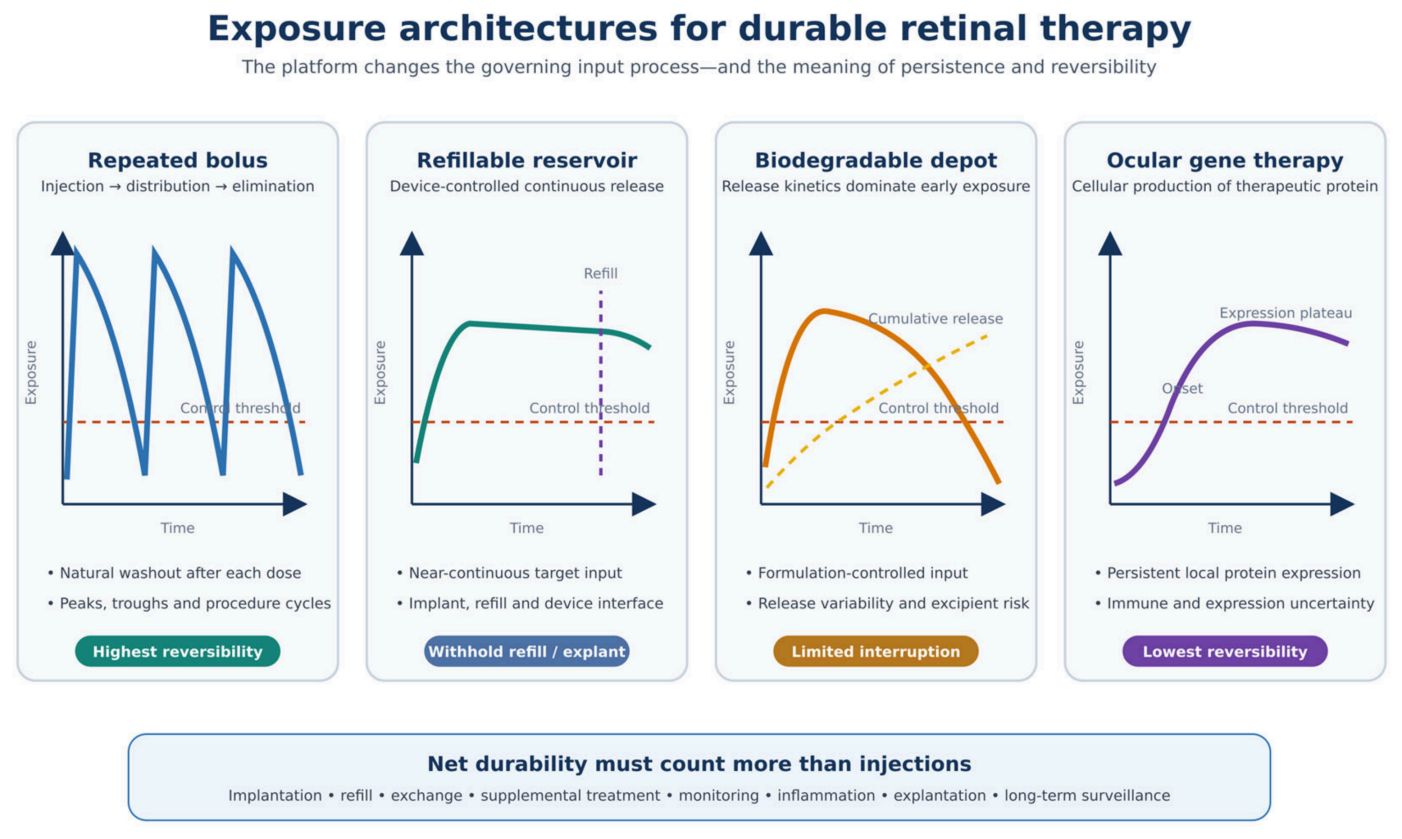
Exposure architectures for durable retinal therapy. Bolus dosing, reservoirs, depots and gene therapy create distinct exposure profiles with different reversibility and surveillance requirements. Curves are conceptual, not quantitative. Solid coloured curves show the conceptual exposure profiles of the labelled platforms; red horizontal dashed lines indicate the control threshold, the purple vertical dashed line indicates reservoir refill, and the yellow diagonal dashed line indicates cumulative depot release.

The next phase of anti-VEGF development is moving beyond incremental extension of the terminal half-life of an injected bolus. Emerging platforms instead redesign the exposure waveform: a refillable reservoir can release a familiar antibody fragment continuously; a biodegradable depot can deliver a small-molecule receptor inhibitor for months; and ocular gene transfer can convert retinal cells into local producers of an anti-VEGF protein. Other approaches alter molecular size, valency or ligand spectrum. These strategies may reduce the number of intravitreal injections, but they also change the meaning of durability. For a bolus biologic, durability is usually the interval to retreatment. For a reservoir, it is the interval to refill or exchange plus the burden of implantation. For gene therapy, it is the stability and safety of transgene expression, with rescue injections remaining possible. A valid comparison must, therefore, integrate efficacy, rescue treatment, procedure burden, reversibility and cumulative ocular risk. Figure 3 compares the exposure architectures created by bolus dosing, refillable reservoirs, biodegradable depots and ocular gene therapy, while Table 7 summarises representative platforms and their principal constraints.

### 8.1. From Bolus Pharmacokinetics to Exposure Architecture

Intermittent intravitreal injections create a high initial concentration followed by an approximately exponential decline. This peak-to-trough pattern can provide strong early target suppression but permits free VEGF to recover once the drug concentration falls below the eye-specific neutralisation threshold. Increasing the dose or affinity delays that crossing point, although it does not remove the oscillation. Sustained-delivery technologies pursue a different objective: to maintain drug concentrations within a therapeutic window for a prolonged period, ideally above the concentration required for disease control but below concentrations that produce local toxicity.

Continuous exposure is not simply a longer version of bolus exposure. A low, stable concentration may reduce recurrent leakage and fluid fluctuation, but its pharmacodynamic adequacy depends on the release rate relative to ongoing ligand production. If disease demand exceeds delivery, activity can recur, even while the device functions normally. Conversely, continuous suppression removes the low-exposure periods between injections and may prolong on-target or off-target effects. The relevant design variables, therefore, include drug loading, surface area, diffusion path, matrix erosion, refill concentration, release kinetics and the anatomical position of the source. The desired product is not the one with the longest nominal release period, but the one that maintains a controllable, biologically adequate exposure with an acceptable safety margin.

This framework separates three forms of long action. Sustained release meters a finite drug store over time. Molecular retention slows distribution or elimination through size, charge, binding or conjugation. Biofactory strategies produce new therapeutic protein within ocular cells. These mechanisms have different failure modes: depletion or device malfunction for reservoirs; heterogeneous tissue retention for molecular constructs; and variable or persistent expression for gene therapy. They should not be grouped under a single undifferentiated label of extended durability.

### 8.2. Refillable Intraocular Reservoirs: Continuous Delivery with a Serviceable Drug Store

The ranibizumab port delivery system (PDS) provides the most mature demonstration that delivery architecture can extend clinical control without changing the therapeutic protein. The permanent, non-biodegradable implant is positioned through the pars plana and contains a concentrated formulation of ranibizumab. The drug is released passively into the vitreous through a porous element and the reservoir can be refilled and exchanged in the clinic. The platform, therefore, decouples the molecular identity of ranibizumab from the peak-and-decay profile of monthly injection.

In the randomised phase III Archway trial, PDS 100 mg/mL with fixed refill-exchange procedures every 24 weeks produced visual outcomes equivalent to monthly ranibizumab in previously responsive neovascular age-related macular degeneration; 98.4% of PDS-treated eyes did not require supplemental treatment before the first scheduled refill [79]. Population pharmacokinetic modelling subsequently supported continuous ocular delivery and showed that serum concentrations remain much lower than after monthly intravitreal injection, although serum exposure is an indirect measure of the macular concentration [80]. The portal extension suggests maintenance of visual and anatomical outcomes over approximately 4 years in continuing participants [81]. In diabetic macular oedema, Pagoda similarly found that PDS every 24 weeks achieved visual outcomes comparable to monthly ranibizumab through the primary analysis period [82]. These results establish sustained target suppression across more than one retinal vascular phenotype.

The PDS also illustrates why injection avoidance is not equivalent to burden elimination. Implantation requires vitreoretinal surgery, while refills require a device-specific technique and conjunctival access. Adverse events associated with the implant–tissue interface, including vitreous haemorrhage, conjunctival erosion or retraction, endophthalmitis and device-related complications, are qualitatively different from those of routine injection and were more frequent in the device arm of Archway [79]. Surgical refinements and device updates can reduce risk, but the benefit–risk calculation remains patient-dependent. The molecular advantage is continuous, refillable and partly reversible delivery; the clinical price is a permanent implant and a procedural safety domain that conventional pharmacokinetic comparisons do not capture.

A refillable reservoir has an important conceptual advantage over gene therapy: exposure can be serviced. The formulation can be replenished, supplemental intravitreal treatment can be given, and the implant can be removed if necessary, although explantation is a surgical procedure. Future reservoirs could, theoretically, accommodate different biologics or concentrations, but compatibility, stability, release performance and regulatory requirements would need to be demonstrated for each payload. Platform flexibility should, therefore, be distinguished from approved interchangeability.

**Table 7:** Selected emerging molecular and delivery platforms for durable control of retinal vascular disease.

Platform and Example	Durability Mechanism	Clinical Evidence/Status (18 August 2026)	Principal Advantage	Principal Constraint
Refillable reservoir: ranibizumab PDS	Passive continuous release of concentrated ranibizumab from a permanent implant; refill-exchange restores the drug store.	Randomised phase III evidence in nAMD and DMO supports 24-week refill cycles; long-term extension data available [79,80,81,82].	Uses a validated biologic; serviceable, refillable and partly reversible exposure.	Implant surgery, refill technique and device/conjunctival adverse events.
Bioerodible TKI insert: vorolanib (EYP-1901/DURAVYU)	Matrix-controlled, prolonged intraocular release with intracellular VEGF-receptor inhibition.	Phase I published; phase II completed; sponsor-reported LUGANO topline data did not meet the prespecified primary endpoint in the full dataset, while LUCIA remained ongoing at the search cutoff [83,84,85,86,87].	Compact payload and potential six-month repeat administration.	Need for peer-reviewed phase III data, confirmation from LUCIA, repeat-dose characterisation and long-term retinal safety.
Bioresorbable TKI hydrogel: axitinib (AXPAXLI/OTX-TKI)	Hydrogel-controlled axitinib release sustains VEGF-receptor blockade after intravitreal administration.	Sponsor-reported positive SOL-1 phase III results; repeat-dose SOL-R comparison remains ongoing [88,89,90].	Potential prolonged control after a routine intravitreal procedure.	Initial pivotal comparator was a single aflibercept dose; full peer-reviewed and repeat-dose evidence is awaited.
Subretinal AAV biofactory: ABBV-RGX-314	AAV8-mediated production of an anti-VEGF-A antigen-binding fragment by transduced ocular cells.	Phase I/IIa results published; active-controlled pivotal trials ongoing [91,92,93].	Potential multi-year protein production after one administration.	Vitrectomy/subretinal surgery, variable expression, pigmentary changes and limited reversibility.
Intravitreal dual-transgene AAV: 4D-150	Engineered capsid delivers aflibercept plus VEGF-C-directed RNA interference.	Interim phase II data are sponsor reported; two phase III nAMD trials are under way [94,95,96,97].	Office-based route and combined VEGF-family suppression.	Vector immunity, retinal transduction variability, inflammation and no established expression off-switch.
Molecular retention: antibody–polymer conjugates	Large hydrodynamic radius and altered tissue transport slow ocular elimination.	Several late-stage programmes have produced mixed efficacy and durability findings.	No permanent device or gene transfer; familiar injection workflow.	Viscosity, injectability, immunogenicity and possible penetration–retention trade-off.
Pathway expansion: sozinibercept plus anti-VEGF-A	VEGF-C/D ligand trapping is added to VEGF-A suppression.	Positive phase IIb signal did not reproduce in phase III; nAMD programme discontinued [98,99,100].	Mechanistically tests escape through additional VEGF-family ligands.	Added target and injection burden without demonstrated phase III incremental benefit.

Development status is time-sensitive. Company-reported results are identified as such and should be replaced or supplemented by peer-reviewed reports when available. AAV, adeno-associated virus; DMO, diabetic macular oedema; nAMD, neovascular age-related macular degeneration; PDS, Port Delivery System; TKI, tyrosine kinase inhibitor; VEGF, vascular endothelial growth factor.

### 8.3. Sustained Small-Molecule Kinase Inhibitor Depots

Intravitreal depots containing receptor tyrosine kinase inhibitors pursue sustained pathway blockade with molecules much smaller than antibodies. Axitinib and vorolanib inhibit intracellular VEGF-receptor signalling rather than sequestering extracellular ligand. Their high potency and small molecular mass permit a large number of active molecules to be loaded into a compact implant. A biodegradable or bioerodible matrix can then govern release over many months, making formulation kinetics rather than the native free-drug half-life the principal determinant of exposure.

EYP-1901, now developed as a vorolanib intravitreal insert, combines a selective tyrosine kinase inhibitor with a bioerodible sustained-delivery platform. The small phase I DAVIO study reported acceptable tolerability and stable measures of visual and anatomical activity after a single administration in previously treated neovascular age-related macular degeneration [83]. The completed phase II DAVIO 2 programme and the phase III LUGANO and LUCIA trials evaluated or are evaluating whether repeat six-month administration can maintain vision while reducing supplemental anti-VEGF treatment [84,85,86,87]. In sponsor-reported topline results, LUGANO did not meet the prespecified primary non-inferiority endpoint for mean best-corrected visual-acuity change in the full dataset, although secondary analyses indicated fewer supplemental injections and maintained anatomical control; LUCIA remained ongoing at the search cutoff [85,86,87]. These findings are provisional pending peer-reviewed publication and the second phase III result.

AXPAXLI (formerly OTX-TKI) uses a bioresorbable hydrogel to sustain axitinib delivery. In the phase III SOL-1 trial, a single implant was compared with a single aflibercept injection, with protocol-defined rescue available; the sponsor reported superiority for the proportion maintaining vision at week 36 and sustained disease control through 52 weeks [88,89,90]. This result is clinically informative but does not, by itself, establish non-inferiority to repeated on-label aflibercept. The complementary SOL-R trial is designed to assess repeat dosing and an active repeated-treatment comparison [88,89]. Until full peer-reviewed pivotal results are available, numerical claims from company disclosures should be regarded as provisional.

Small-molecule depots raise a distinct safety question: selectivity must be preserved across months of continuous retinal exposure. VEGF receptors participate in physiological endothelial signalling; kinase inhibitors may inhibit additional targets at higher local concentrations. Release bursts, incomplete erosion, particle-related inflammation, vitreous floaters and direct effects on retinal pigment epithelium or neural retina must, therefore, be characterised with spatially resolved toxicology and long-term imaging. The absence of systemic toxicity at an ocular dose does not establish a wide retinal therapeutic index. Repeatability is equally important; a six-month first dose is not a durable treatment strategy unless the second and subsequent administrations have predictable exposure and do not cause depot accumulation or increasing inflammation.

### 8.4. Ocular Gene Therapy as an In Situ Anti-VEGF Biofactory

Gene therapy removes the finite drug reservoir by delivering genetic instructions for local therapeutic-protein production. Most programmes use an adeno-associated virus (AAV) vector carrying a genetic sequence that encodes an anti-VEGF protein, together with promoter and regulatory elements that control expression. The vector capsid, dose, route and target cell population determine transduction, while the cassette determines what is produced and at what approximate level. The clinically relevant pharmacokinetic variable is no longer the elimination of an injected protein alone; it is the time course and inter-eye variability of transgene expression superimposed on the disposition of the secreted product.

Subretinal ABBV-RGX-314, also known as surabgene lomparvovec, uses an AAV8 vector encoding a ranibizumab-like anti-VEGF-A antigen-binding fragment. In an open-label phase I/IIa dose-escalation study of 42 previously treated eyes, higher-dose cohorts showed sustained intraocular protein production, maintenance of vision and anatomy, and marked reductions in supplemental anti-VEGF injections over 2 years; one severe vision-loss event associated with macular pigmentary change and dose-related peripheral pigmentary changes indicate that the therapeutic window must be defined [91]. The ATMOSPHERE and ASCENT pivotal trials are evaluating one-time subretinal delivery against active anti-VEGF comparators [92,93]. Subretinal administration can efficiently transduce retinal pigment epithelium but requires vitrectomy and retinotomy, coupling molecular durability to surgical expertise and perioperative risk.

Intravitreal gene delivery is procedurally simpler but must overcome dilution, neutralising antibodies and the internal limiting membrane as a physical barrier to retinal transduction. The 4D-150 design uses an engineered intravitreal AAV capsid and a dual cassette encoding aflibercept together with an RNA-interference component directed against VEGF-C [94]. The design aims to broaden suppression across VEGF-A/VEGF-B/placental growth factor through aflibercept while reducing VEGF-C signalling. Company-reported phase II PRISM data describe a reduced supplemental-injection burden with maintained visual and anatomical outcomes; however, the reports remain interim and are not a substitute for masked pivotal comparison [95]. Two phase III 4FRONT trials are evaluating a single intravitreal administration, with pivotal readouts pending [96,97].

Gene therapy can provide multi-year exposure after one administration but this same feature limits dose control: expression cannot currently be titrated with the precision of a refill, and it cannot be readily switched off if inflammation, excessive target suppression or an unexpected protein effect occurs. Vector immunity, anterior-chamber or vitreous inflammation, corticosteroid requirements, transduction heterogeneity and potential loss of expression all complicate the simple phrase ‘one-time treatment’. A rescue injection can supplement inadequate expression but cannot remove excessive expression. Long-term surveillance must, therefore, include ocular inflammation; retinal vasculitis or occlusion; pigmentary change; atrophy; systemic vector shedding; anti-capsid and anti-transgene immunity; and the stability of rescue-free disease control.

### 8.5. Molecular Retention, Multispecificity and Pathway Expansion

A parallel strategy is to redesign the therapeutic molecule while retaining episodic intravitreal administration. Increasing hydrodynamic size through polymer conjugation can slow distribution and ocular elimination, while increasing binding capacity or valency can delay target escape. Antibody–biopolymer conjugates exemplify this approach: an anti-VEGF antibody is attached to a large, highly hydrated polymer intended to prolong intraocular residence without using a device. The engineering trade-offs include injectability at a clinically acceptable volume, viscosity, aggregation, retinal penetration and immunogenicity. A large construct may remain in the vitreous longer yet penetrate diseased tissue less uniformly; measured whole-eye persistence should, therefore, not be equated automatically with effective concentration at the neovascular complex.

Multispecific constructs attempt to address both exposure and biological escape by engaging more than one ligand or pathway. Targets under investigation include VEGF family members, angiopoietin/Tie2 signalling, inflammatory cytokines, integrins and complement components. The rationale is strongest when the additional pathway is active in the intended phenotype and remains rate limiting after VEGF-A suppression. Adding a target can increase molecular size and complexity, change tissue distribution and introduce a second mechanism of toxicity. It may improve anatomical control without extending exposure or extend apparent durability only in biomarker-defined subgroups. Molecular breadth and pharmacokinetic durability are related design dimensions, not synonyms.

The development of sozinibercept (OPT-302) provides a useful caution. This soluble receptor trap was designed to inhibit VEGF-C and VEGF-D when added to VEGF-A blockade. A randomised phase IIb study reported superior mean visual gain for the higher-dose combination with ranibizumab at 24 weeks [98]. However, the phase III COAST and ShORe studies did not achieve their primary endpoints; development in neovascular age-related macular degeneration was discontinued [99,100]. The discrepancy does not invalidate pathway expansion as a strategy; it shows that a strong biological rationale and a positive intermediate trial do not guarantee incremental benefit over an active control. Future programmes should prospectively demonstrate target engagement; identify the subgroup in which the added pathway is limiting; and distinguish improved efficacy from reduced treatment frequency.

### 8.6. Safety, Reversibility and the Cost of Persistent Exposure

Long action changes the temporal structure of risk. A transient concentration-related adverse effect after a bolus may decline as the drug is cleared. The same effect from a depot or transgene may persist and treatment discontinuation may not promptly reduce exposure. Durability should, therefore, be interpreted as safety-adjusted durability—the period of controlled disease achieved without rescue, device intervention, prolonged corticosteroid treatment, sight-threatening inflammation or unacceptable structural change.

Reversibility exists on a continuum. A conventional intravitreal biologic is not immediately removable, but its concentration falls predictably. A biodegradable depot cannot usually be retrieved completely, although release ends as the payload is exhausted. A refillable implant can be emptied, refilled or explanted, but these actions involve procedure-specific risks. AAV-mediated expression is potentially the least reversible; rescue can add treatment, but there is no established clinical off-switch for transduced ocular cells. A molecular off-switch should be distinguished from vector removal: stopping new synthesis does not eliminate existing intraocular protein, which must decline according to its own pharmacokinetics.

Small molecule-regulated expression is feasible experimentally. Doxycycline-responsive AAV systems have achieved repeated control in non human-primate retina [101]; a tetracycline-responsive RNA aptazyme modulated AAV-derived aflibercept and choroidal neovascularisation in mice [102]. A clinically credible design would be default off, retina-cell restricted, dose-responsive and reversible, with low basal leak, rapid off-kinetics and a short-lived retina-penetrant inducer; an independent transgene-UTR sequence targetable by intravitreal antisense oligonucleotide or siRNA could provide an emergency brake. Transcriptional switches add cassette size and may encode bacterial or viral transactivators that increase adaptive immunogenicity. RNA-only systems may reduce that burden but do not remove immunity to the AAV capsid or therapeutic protein, innate nucleic-acid sensing, off-target effects or inducer toxicity. These approaches remain preclinical safeguards rather than validated human protocols.

Persistent VEGF suppression also requires biological surveillance beyond exudation. VEGF supports choriocapillaris and neuronal homeostasis under some conditions, although the clinical relevance of chronic suppression remains uncertain. Macular atrophy, fibrosis and retinal vascular changes are confounded by the natural history of neovascular disease and by previous treatment. Long-term studies should use masked imaging adjudication, fellow-eye and active-control comparisons, exposure or expression measurements and cumulative incidence rather than reporting only treatment-emergent events at a single time-point.

### 8.7. How Emerging Platforms Should Be Compared

A common evidential framework is needed because nominal dosing intervals are not comparable across platforms. The primary efficacy endpoint should remain vision-based and should be tested against an active regimen that reflects contemporary care. Anatomical outcomes should include central subfield thickness, intraretinal and subretinal fluid, haemorrhage and the amplitude of fluid fluctuation. Durability outcomes should report time to first rescue, the rescue-free proportion at prespecified times, the distribution of rescue counts and the interval after repeat administration. For gene therapy, transgene-product concentration and its variability should be linked to rescue and inflammation; for depots and reservoirs, the release kinetics and residual payload should be linked to disease control.

The treatment burden should include every ocular procedure, not only anti-VEGF injections. Implantation, refill-exchange, monitoring visits, corticosteroid administration, device revision, rescue injection and explantation should be counted and weighted by complexity. Patient-reported discomfort, anxiety, recovery time and preference are important because a six-month refill may be valued differently from a one-time surgical procedure or prolonged steroid course. Health-economic analyses should incorporate failure and rescue pathways rather than assume that a long nominal interval automatically lowers cost.

Trial populations also need to reflect the intended use. Enrichment for previously responsive, stable eyes can establish maintenance but may overestimate rescue-free performance in treatment-naive or high-demand disease. Run-in phases that exclude early persistent fluid can reduce variability but limit generalisability. Results should, therefore, be stratified by baseline treatment demand, lesion and fluid phenotype, disease duration and achieved loading response. A platform should be considered meaningfully durable only when it preserves vision and anatomy across the relevant population, reduces total procedure burden, retains an acceptable rescue pathway and maintains a favourable cumulative safety profile.

### 8.8. Synthesis of Emerging Delivery Platforms

Emerging anti-VEGF strategies extend durability by controlling exposure rather than relying solely on the native half-life of a bolus biologic. The PDS demonstrates that a refillable reservoir can sustain a proven molecule for six-month treatment cycles but introduces surgical and device-specific risks. Small-molecule depots offer high payload density and prolonged receptor inhibition, but require detailed evaluation of selectivity, release and repeat dosing. Gene therapy offers the greatest potential duration by producing therapeutic protein in situ, but also the greatest uncertainty in dose control, reversibility and long-term immune safety. Molecular retention and pathway expansion can add value, although the failure of late-stage VEGF-C/D combination trials shows that broader biology must translate into clinically incremental benefit.

Development should, therefore, prioritise controllable persistence. A suitable platform would maintain therapeutic exposure at the macula; adapt to changing disease demands; permit rescue or de-escalation; and minimise cumulative procedural and molecular risk. No current approach fully satisfies all four requirements. The field should resist ranking technologies by the longest advertised interval and instead compare safety-adjusted, rescue-aware and procedure-inclusive durability.

## 9. Knowledge Gaps and Future Research Priorities

Anti-VEGF durability is often discussed as if it were a single property that could be read directly from a molecule, label or dosing schedule. The preceding sections instead show that the observed interval is jointly determined by ocular exposure; binding and target suppression; disease demand; anatomical phenotype; retreatment rules; and the delivery platform. These dependencies have practical consequences for research. A study can appear to demonstrate greater durability because it uses a more tolerant optical coherence tomography threshold, permits fewer assessment visits, enriches for previously stable eyes or assigns a longer interval before biological recurrence is sought. Conversely, a genuinely longer molecular effect can be obscured by conservative rescue rules or by the inclusion of eyes whose residual fluid is not VEGF-driven. Research should, therefore, focus less on rankings based on nominal interval and more on an evidence framework that separates drug, eye, disease and protocol.

The most useful next studies will define durability in testable terms. They should specify what event ends control; where and how exposure is measured; how rescue treatment is handled; and which safety costs accompany the achieved reduction in routine procedures. Regulatory guidance already calls for well-designed randomised comparisons and prespecified efficacy endpoints in neovascular age-related macular degeneration, while the ICH E9(R1) estimand framework requires the clinical question, intercurrent events and target treatment effect to be aligned before analysis [103,104]. A molecular durability agenda should extend these principles to pharmacokinetic–pharmacodynamic sampling, imaging, biomarkers and long-term platform safety. The priorities below are ordered from foundational measurement problems to translation and implementation.

### 9.1. Standardise the Definition and Reporting of Durability

The first gap is semantic. Terms, such as extended dosing, treatment-free, rescue-free, injection-free and sustained control, are frequently used interchangeably, although they describe different quantities. At minimum, reports should distinguish: the maximum interval permitted by the protocol or label; the interval assigned at a particular visit; the interval actually completed without earlier rescue; the time from treatment to first objective evidence of biological reactivation; the duration of measurable target suppression; and, for devices, the interval between refill, exchange or replacement procedures. Gene therapy additionally requires a distinction between stable transgene expression, freedom from supplemental injection and freedom from inflammation treatment. None of these measures alone captures the total patient experience.

The preferred clinical estimand should be the time for which an eye remains controlled under prespecified functional and anatomical criteria without unplanned rescue, accompanied by the cumulative number of all ocular procedures. Rescue is not a nuisance observation to be censored automatically; it is an intercurrent event that can indicate insufficient efficacy, protocol conservatism, intolerance or a temporary complication. The strategy for handling it should match the clinical question [104]. A treatment-policy estimand may ask how vision is maintained in practice when rescue is available. A composite estimand may define failure as rescue, unacceptable vision loss or a safety-driven discontinuation. A hypothetical no-rescue estimand is less clinically intuitive and depends strongly on unverifiable assumptions. Reporting more than one estimand can be appropriate, but each must be named and interpreted consistently.

Visit frequency creates interval censoring. If recurrence can be detected only at scheduled visits, an apparent 16-week duration may mean that biological activity returned at any time after the preceding visit. Trials with different assessment calendars cannot, therefore, be compared by the observed retreatment week alone. Future studies should report the assessment schedule; the interval between the last inactive and first active visit; and sensitivity analyses that account for interval censoring. Home optical coherence tomography or symptom-triggered assessment may narrow this window, although decentralised measurements require validation and can change behaviour by prompting earlier treatment.

Durability reports should present the full distribution rather than only a mean, median or proportion reaching the longest allowed interval. Useful outputs include Kaplan–Meier curves for protocol-defined loss of control, interval-specific transition probabilities, cumulative rescue counts, the proportion with repeated successful extension and reasons for shortening. Functional outcomes, anatomical fluctuation, treatment complications, patient-reported burden and caregiver time should be presented beside the injection number. Consensus imaging nomenclature can improve the comparability of lesion and fluid phenotypes [105], but adoption must be active: a recent evaluation of the macular degeneration core outcome set found no clear improvement in uptake after its publication, with patient-centred domains measured far less often than distance visual acuity [106].

### 9.2. Measure Human Ocular Pharmacokinetics, Pharmacodynamics and Target Engagement

Direct human evidence linking retinal exposure to clinical interval remains unusually sparse. Vitreous sampling is invasive, repeated retinal or choroidal sampling is generally impossible, and serum concentrations represent drug that has already left the eye. Aqueous humour is accessible but is a downstream surrogate whose relation to free drug at the macula depends on diffusion, convection, tissue binding and anterior elimination. Mechanistic modelling has shown how binding stoichiometry and VEGF turnover can be integrated with intravitreal pharmacokinetics [107], while analyses of published data indicate that the anterior route is a major elimination pathway for intravitreal anti-VEGF macromolecules [108]. These models are informative, but identifiability is limited when several biological processes are inferred from a small number of surrogate concentrations.

A prospective human ocular pharmacology programme should be embedded within phase I–III development rather than added after efficacy is known. Sparse but strategically timed aqueous samples can be collected at dosing, early target suppression, the expected exposure threshold and suspected reactivation. Assays should distinguish free drug, total drug, free VEGF-family ligands, drug–ligand complexes and anti-drug antibodies, where technically feasible. The actual molar dose, formulation concentration, injection volume, time since the previous treatment, sampling time, assay lower limit of quantification and handling of values below that limit must be reported. Low-volume aqueous assays demonstrate technical feasibility, but analytical sensitivity does not eliminate the compartment problem [109].

The highest priority is a mechanism-informed population pharmacokinetic–pharmacodynamic model that jointly analyses ocular concentrations, systemic concentrations, imaging activity and rescue. Covariates should include axial length; lens and vitreous status; renal function where relevant; baseline ligand burden; lesion or oedema phenotype; inflammatory markers; and prior treatment intensity. The model should estimate uncertainty in the unobserved retinal concentration rather than present a single simulated curve as fact. External validation must occur in a separate population and, ideally, with a different dosing regimen. A model becomes translationally useful only if it predicts prospectively which eyes will lose control, identifies a modifiable dosing or delivery parameter or supports a safer reduction in monitoring.

Repeated-dose studies are especially important. Single-dose half-life estimates may not describe a reservoir at steady state, a biodegradable depot after repeat administration or gene-derived protein whose expression changes over months. Studies should test accumulation, time-dependent clearance, immunogenicity, release-rate drift and the relation between exposure variability and rescue. For sustained platforms, release testing in vitro, ocular concentrations in vivo and clinical control should be connected through a prespecified model. This linkage would permit a failed interval to be classified as inadequate release, insufficient tissue distribution, high target production or pathway mismatch rather than labelled generically as non-response.

### 9.3. Resolve Spatial Exposure at the Retina–RPE–Choroid Interface

Whole-eye or vitreous persistence can overstate molecular availability at the site of disease. Anti-VEGF agents must move from the vitreous through the internal limiting membrane and neural retina, or access subretinal and sub-RPE compartments, before they can neutralise the ligand close to a neovascular complex. A larger or more strongly retained construct may have a favourable vitreous half-life while penetrating tissue more slowly. Conversely, tissue or matrix binding can create a local reservoir that is not visible from aqueous measurements. Spatial distribution is, therefore, a co-primary molecular determinant of durability, not a secondary formulation detail.

Preclinical development should combine quantitative whole-eye pharmacokinetics with spatial methods such as autoradiography, immunohistochemistry, mass-spectrometry imaging or validated fluorescent labelling. Measurements should distinguish intact active drug from degraded or dissociated labels and should be made in disease-relevant tissue, not only healthy young animals. Species differences in vitreous volume, retinal barriers, Fc receptor biology and ocular clearance must be carried into translational uncertainty. The aim is to construct concentration–time maps for vitreous, retina, retinal pigment epithelium and choroid, and then to test whether the mapped free concentration exceeds a locally defined pharmacodynamic threshold.

Human confirmation will, necessarily, be opportunistic. Samples obtained during clinically indicated vitrectomy, device explantation or enucleation can provide rare tissue-level anchors if time since dose and tissue location are documented. Imaging may offer non-invasive complementary information: leakage, fluid compartments, choriocapillaris flow and lesion morphology can be registered spatially and linked to model-predicted exposure. These approaches should not be described as direct drug measurements, but they can reject models that predict adequate macular exposure while activity repeatedly recurs in a specific compartment. A long-term goal is a validated ocular digital twin that combines anatomy, molecular transport and disease demand; its first purpose should be experimental design and hypothesis testing, not autonomous clinical dosing.

### 9.4. Develop Biomarkers for Precision Durability, Not Merely Response

Most proposed retinal biomarkers predict a short-term anatomical response or visual change. Precision durability requires a different target: the probability that a specific eye will remain controlled for a defined interval under a defined regimen. Candidate biomarkers should discriminate at least four mechanisms: rapid loss of ocular exposure; unusually high VEGF production; leakage sustained by non-VEGF pathways; and structural fluid that does not represent active permeability. A single baseline cytokine concentration is unlikely to make these distinctions. Serial changes after treatment, coupled to imaging and pharmacokinetic measurements, are more informative because they reveal target engagement and the tempo of biological recovery.

A discovery study should collect aqueous proteomics or targeted ligand panels, optical coherence tomography and angiography features, systemic variables, genotype where justified, and the exact time to protocol-defined reactivation. Pre-analytical controls are necessary because the total protein, lens status, pupil dilation, sampling volume, blood contamination, storage and assay platform can alter the measured profile [69,70,71,72]. Biomarker development should separate discovery, locked-model validation and clinical-utility testing. Cross-validation within a small single-centre cohort is not external validation; a model should be calibrated in different populations, scanners and laboratories; its incremental value over a simple clinical history should be quantified.

The clinical benchmark is not a high area under the receiver-operating-characteristic curve. A biomarker must improve a decision. Prospective trials could randomise eyes at the predicted low risk of recurrence to biomarker-guided extension versus standard treat-and-extend, with non-inferior vision and fewer visits as co-primary objectives. High-risk predictions should be tested as to whether a higher dose, different molecular format, sustained platform or alternative-pathway therapy actually changes the outcome. Decision-curve analysis, calibration plots, false reassurance rates and subgroup performance are required. Algorithms should remain interpretable enough to show whether a recommendation arises from exposure, target or structural features; otherwise, a useful association may be mistaken for a molecular mechanism.

### 9.5. Use Comparative Designs That Isolate Molecular and Platform Effects

Comparative durability trials should change one principal variable at a time. A molecule cannot be credited with a longer biological effect when its arm also receives a different loading schedule, fluid tolerance, assessment cadence or rescue algorithm. Active-comparator trials should, therefore, use harmonised monitoring and the same prespecified disease-activity criteria whenever scientifically and ethically possible. If regimens must differ, the estimand should state whether the comparison concerns the assigned strategies, the intrinsic products under a common schedule or the realised care pathway. Maximum labelled intervals are not valid comparative endpoints because they are imposed design limits rather than observed biological quantities.

An interval-challenge design can be particularly informative after a common initiation or loading phase. Eyes that achieve control are randomised to molecularly distinct treatments, then extended under identical rules with masked image grading. The primary outcome is the time to confirmed reactivation or rescue, with visual non-inferiority and safety safeguards. A randomised-withdrawal component may help to estimate the duration of residual pharmacological effect, although untreated recurrence must be detected promptly and rescued. Enrichment for previously responsive or stable eyes improves efficiency but narrows generalisability; results should identify the run-in selection fraction and compare enrolled with excluded patients.

Sustained-delivery and gene-therapy trials need platform-appropriate comparisons. A reservoir should be compared with optimised repeated injection and should count implantation, refill, exchange, supplemental injection, explantation and unscheduled visits. A depot programme must demonstrate predictable second and subsequent administrations, because a successful first release cycle does not establish a repeatable chronic strategy. Gene therapy requires an active comparator, prespecified rescue and analyses that do not treat supplemental injection as missing data. Expression, corticosteroid treatment and inflammation should be analysed alongside freedom from anti-VEGF rescue. FDA guidance for retinal gene therapy addresses product development, preclinical testing and clinical-trial considerations, reinforcing the need to evaluate the complete vector–route–cassette product rather than the encoded protein alone [110].

Pathway-expansion studies should be biomarker-informed and mechanistically staged. Before exposing large populations to a second target, early trials should demonstrate intraocular target expression, target engagement and an exposure–response relationship. Factorial designs can separate the contribution of the added pathway from changes in anti-VEGF dose. An anatomical improvement without longer control should not be marketed as durability; a phase II signal must not be assumed to validate the mechanism after a negative phase III programme. The sozinibercept experience discussed in Section 8 [98,99,100] illustrates why replication, adequate active control and a prespecified molecular hypothesis are indispensable.

### 9.6. Establish Long-Term Safety, Reversibility and Cumulative-Risk Frameworks

Extending exposure changes the temporal structure of risk. Repeated injections create many short procedural risks; a permanent device creates an enduring tissue interface; a biodegradable depot creates prolonged local exposure to drug and excipient; and gene transfer may produce protein for years. Safety comparisons must, therefore, use a common unit that includes both event severity and time at risk. Reporting events per injection can make a device or gene therapy appear artificially favourable, while reporting only the proportion of patients with any event can obscure repeated injection procedures. Cumulative incidence, recurrent-event analyses and procedure-specific denominators should be presented together.

The principal ocular uncertainties include chronic inflammation; retinal vasculitis or vascular occlusion; sustained intraocular pressure elevation; cataract; endophthalmitis; device erosion; vitreous haemorrhage; retinal pigment epithelial or choriocapillaris effects; fibrosis and macular atrophy. These outcomes have different latencies and biological plausibility across platforms. Long-term imaging should include standardised fundus photography, optical coherence tomography, autofluorescence and angiographic or flow measures where indicated, with masked adjudication of atrophy, fibrosis and vascular events. Analyses should account for baseline disease-associated atrophy rather than attributing every late structural change to treatment or, conversely, assuming that chronic VEGF suppression is neutral.

Reversibility should be treated as a measurable product attribute. For a bolus injection, it is largely governed by elimination. For a refillable device, it includes the feasibility and consequences of withholding refill or explanting the implant. For a depot, it includes whether exposure can be interrupted and how long the material persists. For gene therapy, it includes the management of inflammation, availability of rescue, and the absence or presence of a validated means with which to reduce transgene expression. A proposed off-switch cannot be considered clinically useful until its activation, kinetics, tissue reach and safety have been demonstrated in the same ocular context.

Gene-therapy programmes require surveillance beyond the primary efficacy period because long-acting biological changes can produce delayed adverse events. FDA guidance describes extended follow-up as integral for products with relevant long-term risks [111]. Retinal programmes should prespecify the duration; retention strategy; systemic and ocular assessments; pregnancy considerations where relevant; immunological sampling; and linkage between pivotal trials and long-term cohorts. Similar discipline is warranted for permanent devices, even when they are regulated through a different pathway. Registries should retain active comparator groups and capture product version, surgical technique and operator learning; otherwise, improvements after design modifications may be confused with selection or calendar-time effects.

### 9.7. Build Inclusive, Pragmatic and Patient-Centred Evidence

Durability may provide its greatest benefit to patients who cannot sustain frequent care; however, these patients are often the least likely to enter or remain in intensive trials. Eligibility criteria that exclude bilateral disease, vitrectomised eyes, major systemic comorbidity, unstable access to transport or prior treatment irregularity can produce a pharmacologically clean population that underrepresents the intended clinical use. Racial and ethnic representation is also incomplete: an analysis of almost 10,000 participants in diabetic macular oedema and retinal vein occlusion trials found that enrolment commonly did not reflect the 2010 United States population and that White participants tended to be overrepresented [112]. Representation should be evaluated against disease burden and local catchment populations, not used as a purely descriptive table.

Future pivotal programmes should recruit across continents, regions and care settings, report social determinants that influence follow-up, and prespecify subgroup analyses only when adequately powered or mechanistically justified. Important populations include individuals with diabetes and renal disease, prior vitrectomy, high baseline treatment demand, bilateral active disease, very old age and limited caregiver support. Bilateral treatment creates correlated outcomes but also reflects real procedural burden; excluding the fellow eye from burden-accounting can misrepresent the benefit of a durable strategy.

Pragmatic trials and registry-randomised studies can test whether molecular durability survives ordinary practice. They should capture missed visits, treatment delay, home monitoring, clinician discretion, switches, supplemental injections and loss to follow-up, rather than analysing only adherent completers. Linkage to imaging repositories and electronic records can support large exposure–outcome studies but confounding by indication is substantial: clinicians preferentially extend stable eyes and switch difficult eyes. Target-trial emulation, negative controls and sensitivity analyses can reduce but not abolish this bias. Randomisation remains necessary for causal claims about products.

Patient-centred burden must be measured directly. Treatment days involve travel, waiting, anxiety, discomfort, accompaniment and time away from work or caregiving; a Norwegian survey confirmed substantial and variable burden surrounding intravitreal injections [113]. A credible durability endpoint should, therefore, include patient and caregiver time, treatment anxiety, clinic visits, home-monitoring workload, surgical recovery and out-of-pocket cost. A six-month refill that requires surgery or several monitoring visits is not equivalent to six months without contact. These outcomes should complement, not replace, visual and anatomical safety.

### 9.8. Create Reproducible Data and Modelling Infrastructure

Molecular durability research generates heterogeneous data: concentration–time measurements; ligand assays; vector and device characteristics; longitudinal imaging; rescue decisions; and patient-reported outcomes. Reuse is difficult when units, preprocessing and outcome definitions are not shared. Data packages should include de-identified individual-participant timelines; exact dose and treatment dates; visit windows; reasons for rescue; assay methods and lower limits; image-acquisition metadata; segmentation definitions; missingness indicators; and adverse-event timing. The FAIR principles—findability, accessibility, interoperability and reusability—provide a useful minimum framework [114]; however, accessibility must be implemented through governed repositories and usable metadata rather than as a statement that data are available on request.

Code and model transparency are equally important. Population pharmacokinetic–pharmacodynamic analyses should release equations, parameter priors, covariate selection rules, diagnostics and executable code where intellectual-property and privacy constraints permit. Imaging and biomarker models should preserve a locked version, disclose training-set composition and publish external calibration. A model that is repeatedly refitted to each new dataset cannot demonstrate transportability. Negative and inconclusive studies should also be deposited, particularly when a plausible molecular target or delivery platform fails; publication restricted to successful extension experiments exaggerates the apparent maturity of the field.

A shared benchmark dataset could accelerate progress without dictating a single model. It should include multiple diseases, products and dosing patterns; adjudicated activity labels; raw and processed optical coherence tomography; selected molecular measurements; and a long enough follow-up to observe repeated extension and failure. A blinded challenge could compare models on calibration, time-dependent prediction, subgroup fairness and decision value. Governance must protect privacy and commercial confidentiality, while ensuring that independent investigators can reproduce headline findings. The objective is not an algorithmic league table, but a common test of whether mechanistic claims generalise.

### 9.9. A Prioritised Translational Roadmap

The priorities are interdependent. Standardised durability estimands are needed before biomarkers or platforms can be compared. Human pharmacokinetic–pharmacodynamic sampling is needed before models can distinguish clearance from target production. Spatial experiments are needed before a long vitreous half-life can be interpreted as retinal exposure. Long-term and inclusive studies are needed before a reduction in injections can be claimed as a net clinical benefit. Table 8 converts these dependencies into a practical roadmap. The proposed translation criteria are deliberately demanding. Association is not enough; each programme should demonstrate that the information or platform improves a prespecified clinical decision while maintaining vision and safety.

## 10. Conclusions

Anti-VEGF therapy has transformed the prognosis of neovascular age-related macular degeneration, diabetic macular oedema and macular oedema secondary to retinal vein occlusion, yet its success has created a chronic delivery problem. The clinical demand for longer treatment intervals is, therefore, legitimate; however, the term durability is often used with insufficient precision. A labelled maximum interval, an interval assigned by a treat-and-extend protocol, a period of molecular target suppression and the time for which an individual eye remains free from rescue are related but non-equivalent outcomes. This review argues that durability should be understood as a time-to-threshold phenotype: the interval ends when declining effective exposure, recovering ligand activity, parallel disease pathways and the anatomical expression of leakage cross a prespecified retreatment threshold.

Within this framework, ocular half-life is important but not determinative. Molecular mass, hydrodynamic radius, charge, binding affinity, valency, dose, formulation and interactions with ocular tissues influence intravitreal distribution and elimination. These properties shape the concentration–time profile; however, the clinically relevant exposure is the free, active concentration at the retina–retinal pigment epithelium–choroid interface rather than persistence in the vitreous as a whole. Target kinetics then determine how that exposure is translated into suppression: VEGF production, ligand–drug stoichiometry, receptor competition and the contribution of VEGF-family or non-VEGF permeability pathways can shorten or extend control without changing the measured terminal half-life. Accordingly, a molecular construct may persist longer yet fail to provide proportionately longer disease suppression if tissue penetration is limited, the accessible dose is insufficient, or the biological driver is not adequately engaged.

Clinical interval is further modified by the eye in which the molecule operates. Vitreous status, ocular anatomy, macular neovascularisation subtype, fluid compartment, ischaemic burden, inflammatory activity and chronic structural damage influence both exposure and the anatomical signal used to trigger retreatment. The same pharmacological state may, therefore, generate different observed intervals in two eyes, or in the same eye under different retreatment rules. Treat-and-extend studies have shown that tolerance of selected stable anatomical findings can lengthen the achieved interval without necessarily compromising vision, demonstrating that protocol design is part of the durability phenotype. This does not make durability subjective; it means that the endpoint must identify the biological and clinical threshold being measured.

Current anti-VEGF agents extend control through different combinations of dose, potency, binding architecture and target spectrum. Newer strategies change the exposure architecture more fundamentally. Refillable reservoirs replace peak-and-trough bolus dosing with continuous delivery; biodegradable depots make formulation-controlled release the dominant kinetic process; and ocular gene therapy converts transduced cells into local producers of an anti-angiogenic protein. Molecular-retention and multispecific approaches seek to prolong residence, increase neutralising capacity or address pathway escape. These platforms cannot be compared fairly by the injection number alone. Implantation, refill, exchange, corticosteroid treatment, supplemental injections, monitoring, surgical complications and the reversibility of exposure must all enter into the calculation. The most durable product is not necessarily the one that remains in the eye for the longest period, but the one that provides controllable target-site exposure with the greatest sustained clinical benefit and the lowest cumulative burden and risk.

Persistent exposure makes this issue more important. A conventional intravitreal bolus is naturally self-limiting, whereas a permanent device or gene-derived protein can maintain pharmacological activity for months or years. Prolonged control may substantially reduce routine procedures, but it also extends the period during which inflammation, device–tissue interactions, excessive pathway suppression or unexpected off-target effects can occur. Reversibility should consequently be treated as a measurable product attribute rather than a qualitative reassurance. The feasibility, timing and consequences of withholding refill, removing a device, managing a depot-related event or modulating transgene-derived expression should be evaluated alongside efficacy. Long-term safety surveillance is not ancillary to durability; it is part of its definition.

Aqueous humour biomarkers, serial optical coherence tomography and mechanism-informed pharmacokinetic–pharmacodynamic models may help to individualise durability. Their immediate purpose is not to produce a universal interval score, but to distinguish why an eye loses control: rapid exposure decline, high VEGF production, inadequate access to the target compartment, activation of another pathway or a structural abnormality that is not pharmacologically reversible. Translation will require careful pre-analytical standardisation, direct acknowledgement of the aqueous-to-retina compartment gap, locked external validation and prospective testing of biomarker-guided decisions. A model that predicts recurrence but does not improve monitoring, product selection or patient outcome remains descriptive rather than clinically useful.

For clinicians, the practical implication is to interpret the achieved interval as information about a drug–eye–disease–protocol system, not as a fixed ranking of molecules. A short interval does not automatically indicate rapid drug clearance, and persistent fluid does not necessarily prove incomplete VEGF neutralisation. Conversely, a long assigned interval does not establish continuous molecular suppression if activity is assessed infrequently. Treatment decisions should integrate longitudinal vision, the compartment and trajectory of fluid, haemorrhage, lesion phenotype, prior interval behaviour, patient capacity and the safety characteristics of the delivery platform. This systems view supports individualisation without abandoning pharmacological rigour.

The field is moving beyond ocular half-life towards engineered exposure. Its next advance will depend on connecting that exposure to spatial target engagement, biological recovery and patient-important outcomes. Durability should ultimately be claimed only when a therapy maintains vision and anatomical control for a prespecified period, reduces the total burden of care, remains acceptably safe over the relevant horizon and preserves a credible response to insufficient or excessive exposure. Defined in this way, durability becomes more than a dosing interval: it is an integrated molecular and clinical property that can guide rational drug design, comparative evaluation and personalised retinal care.

## Figures and Tables

**Table 1:** Operational durability constructs and their minimum reporting requirements.

Construct	Primary Quantity	Compartment or Endpoint	What It Can Support	What It Cannot Establish	Minimum Reporting
Ocular molecular persistence	Drug concentration–time profile or elimination half-life	Specified ocular compartment	Residence and exposure modelling	Target suppression or clinical interval	Species, compartment, dose, analyte, sampling design and model
Molecular PD durability	Duration of free-ligand or pathway suppression	Aqueous humour, vitreous or validated tissue surrogate	Mechanistic target control	Retinal engagement or anatomical control by itself	Assay, lower limit, free/total analyte, baseline and suppression definition
Anatomical durability	Time to prespecified recurrent disease activity	OCT, haemorrhage or angiographic endpoint	Maintenance of structural control	Continuous molecular suppression	Activity rule, grading, visit cadence and handling of stable residual findings
Clinical durability	Time without protocol-defined rescue	Vision plus anatomical and clinical criteria	Achieved interval under a specified regimen	Intrinsic molecular persistence independent of protocol	Loading, interval algorithm, rescue, censoring and full interval distribution
Delivery durability	Duration and shape of local input	Reservoir, depot or transgene-derived exposure	Platform-controlled persistence	Net benefit or reversibility	Implantation, refill, exchange, expression kinetics and supplemental treatment
Net durability	Sustained benefit relative to total burden and risk	Patient and health-system horizon	Comparative clinical value	A single mechanistic cause	All procedures, visits, rescue, adverse events, patient burden and reversibility

OCT, optical coherence tomography; PD, pharmacodynamics.

**Table 2:** Molecular architecture and approximate molar exposure of selected intravitreal anti-VEGF biologics.

Agent	Format	Approximate Mass	Typical Dose	Approximate Molecules Delivered	Molecular Interpretation
Bevacizumab	Full-length bivalent IgG1	149 kDa	1.25 mg	8.4 nmol	Large Fc-containing antibody; off-label ocular use; bivalent VEGF-A binding.
Ranibizumab	Monovalent Fab	48 kDa	0.5 mg	10.4 nmol	Compact Fc-free fragment with affinity-matured VEGF-A binding.
Aflibercept 2 mg	Dimeric VEGFR1/VEGFR2 domain–Fc fusion	115 kDa	2 mg	17.4 nmol	High-affinity receptor trap targeting VEGF-A, VEGF-B and PlGF.
Conbercept	Bivalent VEGFR1-D2/VEGFR2-D3-D4–IgG1-Fc fusion	143 kDa	0.5 mg	3.5 nmol	Receptor-domain trap binding VEGF-A isoforms, VEGF-B and PlGF; includes an additional VEGFR2 domain relative to aflibercept [22].
Brolucizumab	Single-chain variable fragment	26 kDa	6 mg	231 nmol	Very high molar dose and small hydrodynamic size; no Fc.
Faricimab	Asymmetric bispecific IgG	149 kDa	6 mg	40.3 nmol	One VEGF-A-binding arm and one Ang-2-binding arm; engineered Fc.
Aflibercept 8 mg	Same receptor–Fc fusion as aflibercept 2 mg	115 kDa	8 mg	69.6 nmol	Fourfold higher starting molecular exposure without a change in binding protein.

Molar amounts are approximate mass/molecular-mass calculations for drug molecules and do not equal functional binding-site equivalents. Ang-2, angiopoietin-2; Fab, antigen-binding fragment; Fc, fragment crystallisable; IgG, immunoglobulin G; PlGF, placental growth factor; VEGF, vascular endothelial growth factor; VEGFR, VEGF receptor.

**Table 3:** Human ocular pharmacokinetic and molecular pharmacodynamic evidence for selected anti-VEGF agents.

Agent and Dose	Human Ocular Concentration Evidence	Reported or Estimated Ocular Half-Life	Human Molecular PD Evidence	Evidence Class and Principal Limitation
Bevacizumab 1.5 mg *	Prospective cross-sectional aqueous-humour sampling in 30 non-vitrectomised eyes, 1–53 days after injection [30].	9.82 days (aqueous humour)	No comparable longitudinal human aqueous VEGF-suppression dataset establishing a suppression duration.	B. Direct human concentration measurement, but one sample per eye; aqueous rather than vitreous or retina.
Ranibizumab 0.5 mg	Prospective cross-sectional aqueous-humour sampling in 18 non-vitrectomised eyes, 1–37 days after injection [31].	7.19 days (aqueous humour)	Mean aqueous VEGF-A suppression: 36.4 ± 6.7 days (26–69) in nAMD [32]; 33.7 ± 5.1 days (27–42) in DMO [33].	B. Direct human concentration and longitudinal biomarker evidence, obtained in separate cohorts; free aqueous ligand is a surrogate.
Aflibercept 2 mg	Serial aqueous-humour sampling through day 28 in five treatment-naïve nAMD eyes [34].	Approximately 11 days (free aflibercept in aqueous humour)	Mean suppression below assay quantification >71 ± 18 days [35]; comparative mean 67 ± 14 days (49–89) [36].	A/B. Rare serial human PK plus longitudinal biomarker evidence, but PK sample was only five eyes and PD remains aqueous/assay-dependent.
Aflibercept 8 mg	No independently established serial human ocular half-life. Higher-dose exposure is inferred from the same molecule and dose-dependent PK/PD models [29,37].	Not directly established; model estimates are approximately 7–8 days and should not be treated as measured clearance.	Longer time above an effective threshold is predicted from the fourfold higher dose; no definitive longitudinal human aqueous target-suppression duration.	C/D. Model- and trial-supported exposure inference; greater dose does not itself prove a longer intrinsic half-life.
Conbercept 0.5 mg	No direct serial human ocular concentration study identified; published ocular PK is primarily preclinical [38].	Approximately 4.24 days in rabbit vitreous; no direct human estimate [38].	No comparable longitudinal human aqueous free-ligand suppression series identified.	D. Preclinical ocular PK only; animal residence must not be converted directly into a human treatment interval.
Brolucizumab 6 mg	Human cross-sectional nSMOL LC–MS/MS study: 52 eyes, samples concentrated near week 4 [39]. Earlier translational models used preclinical inputs [40].	9.00 days (95% CI 7.51–11.2; human aqueous PPK) [39]; 4.4–5.1 days in earlier translational models [40].	Model-predicted mean VEGF-suppression duration approximately 51 days (48–59) [40]; not a direct human target-suppression series.	B/C/D. Discordant methods; cross-sectional groups differed, early samples were sparse, and below-quantification values were imputed.
Faricimab 6 mg	Human popPK model using 1095 aqueous concentrations (284 patients) and 8372 plasma concentrations (2246 patients) [41].	7.5 days (modelled vitreous-to-aqueous elimination)	PopPK/PD prediction of ≥50% aqueous suppression for approximately 12–14 weeks for Ang-2 and 9–10 weeks for VEGF-A [42].	C. Large human datasets, but vitreous PK and suppression durations are model-derived; aqueous biomarkers, high Ang-2 BLQ frequency, sponsor-supported analysis.

Values are reported as published and are not harmonised across compartments, assays, or models. Evidence classes: A, serial human ocular concentration measurements; B, cross-sectional or longitudinal human ocular measurements; C, human population PK or PK/PD modelling; D, preclinical or mechanistic modelling. * The commonly used off-label bevacizumab dose is 1.25 mg; the cited human aqueous PK study administered 1.5 mg. Ang-2, angiopoietin-2; BLQ, below the limit of quantification; CI, confidence interval; DMO, diabetic macular oedema; LC–MS/MS, liquid chromatography–tandem mass spectrometry; nAMD, neovascular age-related macular degeneration; nSMOL, nano-surface and molecular-orientation limited proteolysis; PD, pharmacodynamics; PK, pharmacokinetics; popPK/PD, population pharmacokinetic/pharmacodynamic; PPK, population pharmacokinetic; VEGF-A, vascular endothelial growth factor-A.

**Table 4:** Clinical translation of selected molecular strategies intended to extend anti-VEGF treatment intervals.

Agent and Molecular Strategy	Pivotal Regimen	Trial-Based Durability Signal	What the Evidence Supports	Principal Qualification
Ranibizumab 0.5 mg VEGF-A Fab benchmark	Monthly dosing in pivotal nAMD trials [1].	Continuous monthly VEGF-A suppression improved and maintained vision.	Establishes the efficacy benchmark for sustained VEGF-A neutralisation.	Monthly dosing tests efficacy, not the longest effective interval.
Aflibercept 2 mg High-affinity VEGF trap; VEGF-A/VEGF-B/PlGF	Every 8 weeks after three monthly doses in VIEW [45].	Visual outcomes comparable with monthly ranibizumab during the fixed phase.	Supports clinically effective 8-week control with fewer injections.	Affinity, dose, target spectrum and regimen differ; mechanism cannot be reduced to half-life.
Conbercept 0.5 mg VEGFR1-D2/VEGFR2-D3-D4–Fc trap	Every 12 weeks after three monthly doses in PHOENIX [46].	Initial visual and anatomical gains were maintained to month 12 under the q12-week protocol.	Supports q12-week maintenance after loading in a China-based delayed-treatment trial.	No contemporary active comparator; PANDA-1/2 were terminated because the desired primary endpoint was not met [47,48].
Brolucizumab 6 mg High molar VEGF-A payload in a 26-kDa scFv	Every 12 weeks after loading, permanently shortened to every 8 weeks for activity [49].	At week 92, estimated every-12-week maintenance: 45.4% (HAWK) and 38.6% (HARRIER); greater fluid resolution [49].	Shows extended control in a substantial selected subset.	One-way interval shortening and product-specific inflammatory risk affect safety-adjusted durability; the findings do not establish an inherent scFv-class liability [50,51].
Faricimab 6 mg Bispecific VEGF-A/Ang-2 inhibition	Up to every 16 weeks in year 1; treat-and-extend every 8–16 weeks in year 2 [18,52,53,54].	Most nAMD patients reached every 12–16 weeks; in DMO, >60% were every 16 weeks and approximately 80% were every 12 weeks or longer at week 96 [53,54].	Validates extended dual-pathway regimens in nAMD and DMO.	Ang-2 mediation is plausible, not proven; effects may vary between acute ischaemic sprouting and chronic vascular stabilisation [17,20].
Aflibercept 8 mg Fourfold dose of the established VEGF trap	Every 12 or 16 weeks after loading; interval modification and extension up to 24 weeks in year 2 [55,56,57,58].	Week-48 assigned-interval maintenance: 79%/77% in PULSAR and 91%/89% in PHOTON; efficacy sustained through week 96 with fewer injections.	Strong evidence that dose intensification prolongs clinical exposure above an effective threshold.	Does not establish a distinct intrinsic half-life; cross-trial maximum intervals are not directly comparable.

Intervals are reported as achieved within their original trial algorithms and should not be compared as if they were generated by a common treat-and-extend protocol. DMO, diabetic macular oedema; nAMD, neovascular age-related macular degeneration; PlGF, placental growth factor; scFv, single-chain variable fragment; VEGF, vascular endothelial growth factor.

**Table 8:** Prioritised translational roadmap for molecular anti-VEGF durability research.

Priority	Key Question	Recommended Design	Minimum Measurements	Translation Criterion
1. Common durability estimands	What event and time scale define sustained control?	Consensus exercise followed by prospective use in randomised trials.	Visit calendar; last inactive and first active visit; rescue reason; all ocular procedures; vision; fluid compartment; patient burden.	Reproducible estimates across trials using the same regimen and documented handling of rescue and interval censoring.
2. Human ocular PK/PD	Which exposure and target-suppression parameters determine recurrence?	Prospective sparse aqueous and serum sampling embedded in dose-ranging and pivotal studies; external population-model validation.	Free and total drug; ligands and complexes; anti-drug antibodies; assay limits; OCT activity; exact dose and sampling times.	Prospective prediction of loss of control and a validated dosing or monitoring decision superior to clinical history alone.
3. Spatial target-site exposure	Does persistent vitreous drug remain active at retina, RPE and choroid?	Disease-relevant animal mapping with opportunistic human tissue anchors and registered imaging.	Intact active drug by compartment; binding and degradation; anatomy; lesion location; local pharmacodynamic response.	A validated concentration–time map that explains compartment-specific activity and informs molecular or delivery design.
4. Precision biomarkers	Can eyes with rapid clearance, high ligand demand or pathway mismatch be distinguished?	Serial multi-omic and imaging discovery, locked external validation, then biomarker-guided randomised trial.	Pre-analytical covariates; dynamic molecular changes; OCT/OCTA; systemic factors; achieved interval; rescue and harms.	Non-inferior vision and safety with fewer visits or procedures, plus calibrated benefit across relevant subgroups.
5. Fair platform comparison	Is benefit caused by the molecule, regimen or delivery architecture?	Active-comparator trial with harmonised loading, assessment and rescue; repeat-dose evaluation for chronic platforms.	All procedures; rescue; exposure or expression; inflammation; device or depot status; patient-reported burden.	Reduced total burden with maintained function and anatomy under a clinically relevant estimand.
6. Long-term safety and reversibility	What cumulative and delayed risks accompany persistent exposure?	Extended active-control cohorts and product-specific registries with adjudicated events and version tracking.	Inflammation; vascular events; atrophy; fibrosis; IOP; surgical events; expression or device status; reversibility actions.	Stable benefit–risk over multiple years and a demonstrated, feasible response to inadequate or excessive exposure.
7. Inclusive effectiveness	Does durability work in patients with the greatest treatment burden?	Multi-region pragmatic or registry-randomised studies with retention support and prespecified representativeness metrics.	Disease and ocular history; demographics; social determinants; adherence; bilateral procedures; caregiver time and cost.	Consistent net benefit across intended populations without widening access or outcome disparities.
8. Open, reproducible evidence	Can independent groups reproduce and transport molecular claims?	Governed individual-participant repository, benchmark dataset and blinded model validation.	Timelines; assay SOPs; image metadata; code; model versions; missingness; negative studies; data provenance.	Independent reproduction, external calibration and predefined performance in a held-out population.

The numerical ordering indicates dependency and near-term priority rather than scientific importance. IOP, intraocular pressure; OCT, optical coherence tomography; OCTA, optical coherence tomography angiography; PK/PD, pharmacokinetics/pharmacodynamics; RPE, retinal pigment epithelium; SOP, standard operating procedure.

## Data Availability

No new data were created or analyzed in this study. Data sharing is not applicable to this article.
